# Performance of cementitious systems containing calcined clay in a chloride-rich environment: a review by TC-282 CCL

**DOI:** 10.1617/s11527-024-02426-7

**Published:** 2024-07-23

**Authors:** Yuvaraj Dhandapani, Alisa Machner, William Wilson, Wolfgang Kunther, Sumaiya Afroz, Taehwan Kim, Franco Zunino, Shiju Joseph, Fragkoulis Kanavaris, Arnaud Castel, Karl-Christian Thienel, Edgardo F. Irassar, Shashank Bishnoi, Fernando Martirena, Manu Santhanam

**Affiliations:** 1 School of Civil Engineering, University of Leeds, Leeds, UK; 2 Technical University of Munich, TUM School of Engineering and Design, Department of Materials Engineering, Professorship for Mineral Construction Materials, Munich, Germany; 3 Université de Sherbrooke, Sherbrooke, QC Canada; 4 Technical University of Denmark, Kongens Lyngby, Denmark; 5 Bangladesh University of Engineering and Technology, Dhaka, Bangladesh; 6 University of New South Wales, Sydney, Australia; 7 ETH Zurich, Zurich, Switzerland; 8 University of Cambridge, Cambridge, UK; 9 Technical Specialist Services, Materials, Arup, London, UK; 10 University of Technology Sydney, Sydney, Australia; 11 Universität Der Bundeswehr Munich, Neubiberg, Germany; 12 Universidad Nacional del Centro de La Provincia de Buenos Aires, Tandil, Argentina; 13 Department of Civil Engineering, IIT Delhi, New Delhi, Delhi India; 14 UCLV Cuba, Santa Clara, Cuba; 15 Department of Civil Engineering, IIT Madras, Chennai, India

**Keywords:** Calcined clay, Chloride exposure, Diffusion, Migration, Field exposure

## Abstract

In this review by TC- 282 CCL, a comprehensive examination of various facets of chloride ingress in calcined clay-based concrete in aggressive chloride-rich environments is presented due to its significance in making reinforced concrete structures susceptible to chloride-induced corrosion damages. The review presents a summary of available literature focusing on materials characteristics influencing the chloride resistance of calcined clay-based concrete, such as different clay purity, kaolinite content and other clay minerals, underscoring the significance of pore refinement, pore solution composition, and chloride binding mechanisms. Further, the studies dealing with the performance at the concrete scale, with a particular emphasis on transport properties, curing methods, and mix design, are highlighted. Benchmarking calcined clay mixes with fly ash or slag-based concrete mixes that are widely used in aggressive chloride conditions instead of OPC is recommended. Such comparison could extend the usage of calcined clay as a performance-enhancing mineral admixture in the form of calcined clay or LC2 (limestone-calcined clay). The chloride diffusion coefficient in calcined clay concrete is reported to be significantly lower (about 5–10 times in most literature available so far) compared to OPC, and even lower compared to fly ash and slag-based concrete at early curing ages reported across recent literature made with different types of cements and concrete mixes. Limited studies dealing with reinforcement corrosion point out that calcined clay delays corrosion initiation and reduces corrosion rates despite the reduction in critical chloride threshold. Most of these results on corrosion performance are mainly from laboratory studies and warrant field evaluation in future. Finally, two case studies demonstrating the application of calcined clay-based concrete in real-world marine exposure conditions are discussed to showcase the promising potential of employing low-purity calcined clay-based concrete for reducing carbon footprint and improving durability performance in chloride exposure.

## Introduction

Chloride-induced corrosion is one of the major durability concerns for reinforced concrete structures [1, 2], specifically for large concrete infrastructure [3, 4]. It leads to significant maintenance and repair costs and also increases the demand for cement-based materials for the repair and replacement of concrete to maintain the intended service life [5, 6]. It is well understood that cementitious systems with reactive supplementary cementitious materials (SCMs), including fly ashes, slags, silica fume and calcined clays, could have very good resistances against chloride ingress and chloride-induced corrosion based on a large volume of literature on traditional SCMs in the past decades [7–11]. However, calcined clay encompasses a large family of materials with different properties, including purity, mineralogy and reactivity, and is often used in composite multi-component cementitious systems (i.e., in binary forms or in combination with other resources), which could affect physical, chemical and mineralogical characteristics of hydrated cementitious matrix that control the performance [12–21]. Although there is documented evidence of calcined clays being studied and utilised as cement substitutes since the 1950s, large volumes of fly ash availability from thermal power plants led to the wide adoption of fly ash based blended cements in the past few decades [22–29]. Clays belong to the group of phyllo-silicates, which consist of alternating layers of octahedrally coordinated aluminium and tetrahedrally coordinated silicon.

Clays are complex with various levels of purities, clay mineralogy, and differences in associated minerals, and a detailed summary of the composition and reactivity of clays are discussed in [30–32]. In the context of this paper, low-purity clays are classified as clays that are not commercial metakaolin, henceforth will include clays with lower kaolinite content in them and/or with other impurities or associated minerals and/or mixed clays with more than one dominant clay mineral. Kaolinitic clays, known as 1:1 clays based on Si:Al ratio, is known to be more reactive in comparison to 2:1 clays, such as illite, smectite, etc. [16, 18]. Previous reviews from RILEM TC 282-CCL summarised a range of information related to calcined clay and calcined clay-based cement/concrete, including the mineralogy and availability of clays [30, 31], activation treatment for clays [33], hydration and blend design [34], and fresh and hardened concrete properties [35–37]. The use of calcined clay is acknowledged as natural calcined pozzolana in many existing standards worldwide [38–42] and a more detailed review on the standardisation of calcined clay was carried out by this TC, highlighting potential routes for adopting calcined clay for low clinker cement and calcined clay as manufactured pozzolana [43].

The mineralogy of the calcined clays used as SCMs has several effects on the hydrated phase assemblage and microstructure of hydrated cementitious systems in addition to their workability and strength development [16, 32, 34, 35, 37, 44]. Clay sources including alternative clay types [25, 45–52] and mixed clays containing kaolinite with other clay minerals from sources such as mine tailing, excavation soil, etc. [53–58] and marine clays from excavation and dredging [59, 60] are also increasingly explored due to their wide availability. The chemical and mineralogical composition of the clays used as raw materials influences the phase assemblage formed by the reaction of the calcined clays in the blended Portland cementitious system [12, 16, 61, 62]. Generally, 1:1 clays are known to be more reactive than 2:1 clays [16, 32, 52]. Kaolinite clays are often reported to be more reactive than other clay types, and the higher reactivity, degrees of reaction lead to a denser microstructure [49, 63] and the formation of increased quantities of calcium alumino-ferrite hydrate phases (e.g. AFm phases or AFt phases) [20, 64–66] and an increase in the aluminium incorporation into the C–S–H phase [67, 68]. The type of calcium alumino-ferrite hydrates formed depends largely on the availability of sulfates, carbonates or chloride ions in the pore solution [20, 64, 69–72]. The majority of reports on calcined clays have focused on kaolinitic 1:1 clays and their combination with limestone as SCMs [49, 52, 63, 73, 74]. Although an increased replacement of limestone in these binders will lead to differences in phase assemblages and significant variations in strength and permeability depending on the calcined clay-to-limestone ratio [74–76], higher limestone content may lead to a dilution in the reaction products and may result in reduced performance in some instances [74, 77–81]. Besides calcined clay usage to produce limestone calcined clay cement (LC3), there is also increasing interest in using calcined clay as manufactured pozzolan in the form of LC2 (i.e., limestone calcined clay mixture) directly in concrete production as performance enhancers or mineral admixture to produce concrete with improved resistance to chloride and lower CO_2_ footprint of the concrete [43, 82, 83].

In this review by RILEM TC 282-CCL, the suitability of calcined clay-based concrete for aggressive conditions dominated by chloride ion exposure, like marine environments, de-icing salts and chloride-contaminated groundwater, is highlighted to increase the uptake of calcined clay adoption for concrete used in such aggressive environments. The performance of calcined clay-based concrete for marine and chloride-rich exposure conditions is summarised with respect to two major aspects: (1) understanding the fundamental chemical interaction of chloride with the microstructure (ingress and binding) of calcined clay systems and (2) comparison of the performance of calcined-clay concrete with other blended cement systems. The paper also addresses some of the challenges in assessing chloride resistance and service-life estimation for calcined clay-based concrete. Hence, this comprehensive review would provide scientific background on how the effects of the chemical and mineralogical composition of calcined clays influence both the permeability and the chloride binding to highlight the improved chloride performance of concrete containing calcined clays. Further challenges associated with improving fundamental knowledge on interaction of chloride with the new composite cement based on calcined clays, and the use of existing testing methodologies and guidelines for assessing the suitability and performance of calcined clay concrete for marine exposure based on recent literature and case studies are presented in this review to further the adoption of calcined clay in concrete construction.

## Effect of material characteristics and microstructure on chloride binding and ingress

### Chemical interaction of chlorides

#### Effect of binder composition on chemical and physical chloride binding

**Fig. 1:**
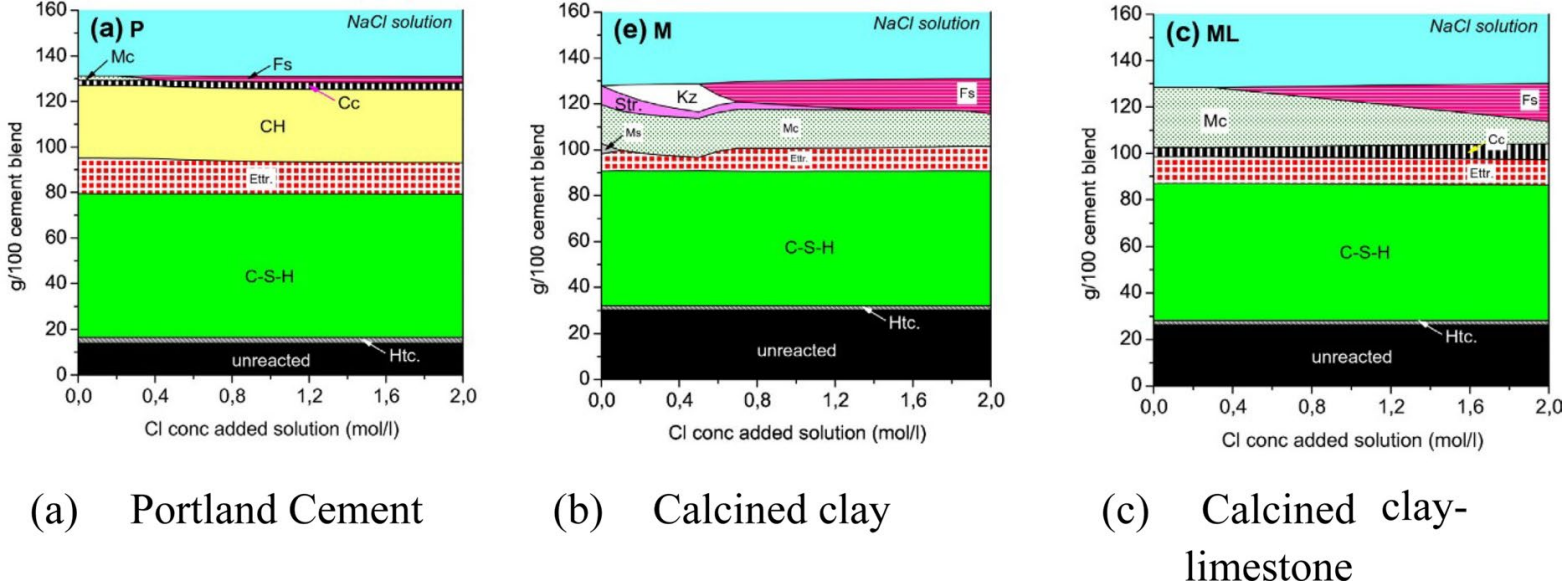
Thermodynamic prediction of the phase assemblage in hydrated OPC (**a**), binary blend of cement with calcined clay (**b**) and a ternary blend of cement, calcined clay and limestone when in contact with chloride solution of increasing concentration [99]. Reprinted from [99] with copyright permission from Elsevier

Kaolinite content of clay is known to influence the amount of alumina rich AFm (Al_2_O_3_–Fe_2_O_3_-monophases) phases formed and the amount of alumina incorporated in the C–A–S–H phase [20, 84]. Similarly, alumina content and reactivity of clay type, i.e., 1:1 or 2:1, is also known to influence alumina rich hydrated phases that could influence the chemical interaction of chloride with the microstructure [16, 18]. While alumina rich SCMs are generally perceived to improve chloride resistance, the extent of calcined clay additions on increasing the chloride binding capacity of hydrated cementitious materials (compared to the reference Portland cement) is still a major discussion in the literature. Chloride binding can manifest in forms of chemical binding that involves transformation of phases in hydrated cement matrix to chloride bound phases and physical binding, in terms of surface adsorption of chloride ions on hydrated phases [85–87]. Chemical binding of chlorides from the exposure solution by cement hydrates involves AFm phases. AFm phases denote a group of layered structure aluminate hydrates with the general formula C_4_(A,F)X_2_·yH, where X represents one mole of a monovalent (OH^−^, Cl^−^) or half a mole of a divalent (SO_4_^2−^, CO_3_^2−^) anion [88–94]. Thus, when exposed to chlorides, AFm phases can incorporate chloride ions in the interlayer and transform into phases such as Friedel’s salt, Kuzel’s salt or solid solutions [88, 89, 93, 94]. This binding is influenced by numerous factors, including the amount of AFm phases, Al content, calcium hydroxide content, pH of hydrated cement’s pore solution and pH of the exposure solution [95]. The formation of Cl-containing AFm phases, like Friedel’s salt, depends on the binder composition and on the chloride exposure solution (concentration and volume). These chloride-containing AFm phases are thermodynamically stable, and consequently their binding in the reaction products can be sustained over time [96–98]. In hydrated cementitious systems containing calcined clays, the amount of AFm phases can be significantly higher compared to plain OPC systems due to the additional Al available to the system from the reaction of calcined clays. This has been shown by means of thermodynamic modelling by Shi et al. [99]. For example, Fig. 1 shows the comparison of phase alterations with increasing chloride ion concentration on the x-axis for OPC, calcined clay paste and calcined clay-limestone paste. As shown in Fig. 1, the AFm and the chloride binding phases in a binary system (Fig. 1B) and a ternary system (Fig. 1C) are much more prevalent than those observed in the plain OPC system. Thus, the maximal chemical binding capacity of these pastes is also expected to be higher compared to plain OPC systems, as reported in several publications [99–101]. However, the AFm phases in real cement pastes can differ from thermodynamic predictions (even after prolonged hydration), because hemicarbonate can be stabilized by sulfates and/or chloride, forming solid solutions [69, 102]. Thus, although the total content of AFm can be higher with calcined clays, the actual chemical binding will depend on the chloride content of the AFm solid solutions [103]. For example, studies found that the amount of Friedel’s salt formed increased from 10 to 25% as the kaolinite content increased from 20 to 50% and reduce thereafter with increasing kaolinite content in the clays [104].

**Fig. 2:**
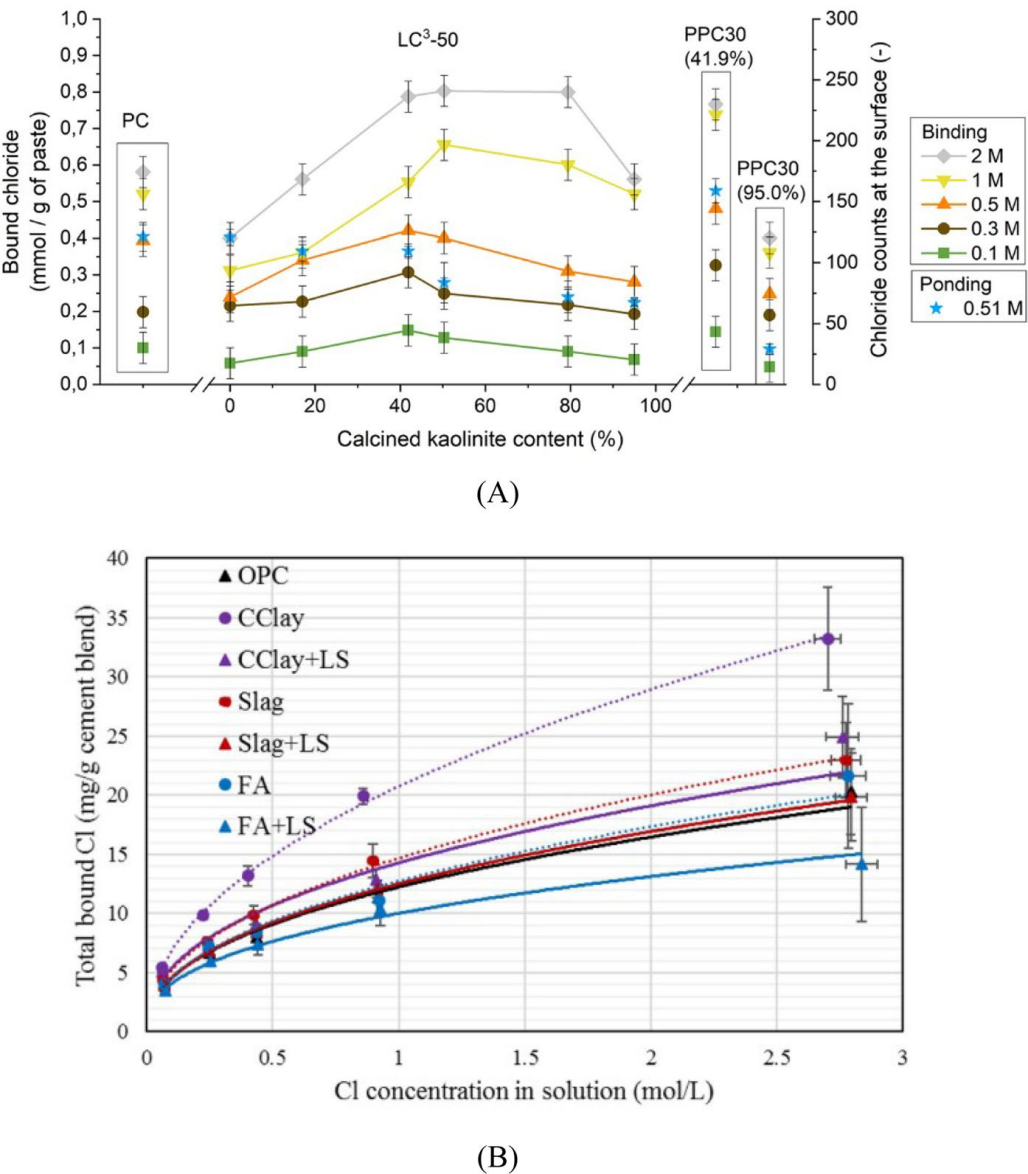
Chloride binding in Calcined clay systems: **A** influence of kaolinite content on chloride binding in PC (plain OPC mixture), LC3 systems using different clays (LC3-50 mixtures with 0% kaolinite content (quartz) to 95 wt% kaolinite content), and two PCC systems (70% OPC and 30% calcined clay mixtures (41.5% and 95.0% kaolinite contents of clays) from [104] and **B** Comparison of binding isotherms in calcined clay and calcined clay-limestone blends compared with OPC, fly ash and slag blends from [100]. Reprinted from [104] with copyright permission from Elsevier

Chloride binding is experimentally measured and presented in the form of chloride binding isotherms made of a plot of bound chlorides (y-axis) at different concentrations of chloride solution (x-axis). This is studied by exposing a few grams of powder hydrated cement to various concentrations of chloride solution till equilibrium is attained. Finally, the concentration of chloride bound in the cement can be measured directly on cement paste or on exposure solution (i.e., reduction in the concentration of exposure solution). Figure 2 present chloride isotherms from [100, 104] for various composite cement pastes containing fly ashes, slag and calcined clay with/without limestone. Figure 2A showcases how the kaolinite content of the calcined clay modifies the total binding and shows that maximum binding occurs for calcined clay with kaolinite content between 40 and 80% [104]. Figure 2B demonstrates the variation in binding for composite cements containing calcined clay-limestone, slag-limestone and fly ash-limestone. While the binary calcined clay blend yields improved binding, calcined clay-limestone combination results in similar total chloride binding compared to OPC [100]. Similarly, other studies have shown that the total bound chloride content was not considerably different with the addition of calcined clay or metakaolin [99, 101, 104, 105]. On the other hand, studies on chloride binding with lower addition of metakaolin in the range of 8–30% have shown significant increase in the chloride binding capacity [99, 106]. A more detailed study on clays with different kaolinite content also reported no significant effect of different kaolinite content on chloride binding which could directly explain the performance [104, 105]. Hence, chloride binding cannot be the sole reason for improved chloride resistance with calcined clay-based concretes. Additionally, chloride binding experiments are not standardized and there are no agreed forms of testing, duration, or expression of data which makes it difficult to come up with conclusive arguments on the performance.

Similar to AFm phases, other hydration products, such as hydrotalcite, which belong to the group of layered double hydroxides (LDHs), are able to chemically bind chlorides. Hydrotalcite (e.g. Mg_6_Al_2_(OH)_18_3H_2_O) is a magnesium and aluminium containing hydration phase with a somewhat variable composition, which is commonly observed to form upon hydration of cement pastes containing ground granulated blast furnace slag [107, 108]. The formation of hydrotalcite has been reported in cement pastes that contain dolomite [CaMg(CO_3_)_2_] and small amounts of metakaolin [109] and in MgO-calcined clay combinations [110]. The chloride binding capacity of hydrotalcite depends on its Mg/Al ratio [109] besides other ions in the solution [109], and binding in the hydrotalcite phase could vary from very low amounts [103] of chloride binding to quantities similar to per mol of Friedel’s salt [109, 111]. Beside binding of chlorides by AFm or other LDH (Layered Double Hydroxide) phases, chlorides can also be physically bound by C–S–H. This physical binding can be explained by the electric double layer (EDL) theory [112, 113]. When divalent cations, such as Ca^2+^ accumulate in the Stern layer, the originally negative surface charge of C–S–H can be overcompensated [113]. The positively rendered Stern layer leads to the formation of a diffuse layer, in which negatively charged ions, such as chloride (Cl^−^) or hydroxyl (OH^−^), may accumulate [112, 113]. This accumulation of chloride ions in the diffuse layer of the C–S–H is commonly referred to as physical binding of chlorides by C–S–H. The accumulation of anions, i.e., chlorides, in the diffuse layer depends on the original surface charge of the C–S–H, which depends, amongst others, on its Ca/Si and Al/Si ratio [114–119]. The C–A–S–H formed during the pozzolanic reaction of calcined clays is commonly characterized by a lower Ca/Si and higher Al/Si ratio compared to C–S–H formed during clinker hydration [67, 120]. With decreasing Ca/Si or Ca/(Si + Al) ratios, a decrease in the physical binding of chlorides by C–(A)–S–H has been reported [103, 119, 121]. The effect of Al/Si ratio on the chloride binding capacity of C–A–S–H is less clear. While Yoshida et al. [114] showed that the chloride binding of C–A–S–H decreases with increasing Al/Si ratio, Jin et al. [115] showed that C–A–S–H phases overall show higher Cl-adsorption compared to C–S–H when comparing the same Ca/(Si + Al) ratio.

The absolute composition of the C–S–H phase depends on the degree of reaction of the cement and the calcined clay and therefore on their mineralogical composition; the calcination temperature of the clay that influences reactivity also plays a role [49, 52, 63, 122, 123]. It should be noted that due to the pozzolanic reaction of calcined clays, more additional C–S–H is formed, which might disguise the variations in total chloride content bound per mol of C–S–H [99, 119, 124]. Therefore, calcined clays with varying reactivities are reported to result in different quantities of anions physically bound by the C–S–H phase [99, 119, 124]. The role of the EDL (physically bound chlorides) on chloride diffusion is still not well understood nor quantified. This could be a key mechanism to explain the increased chloride resistance of new higher performance binders, like the LC3 as attempted in [125–128].

#### Effect of pore solution composition

The ionic composition of pore solution and its conductivity influence the chloride ingress in cementitious systems by modifying the ionic diffusion in the microstructure of the hydrated cement paste and impacts the binding capacity. The use of most SCMs causes a slight reduction in the pH of the pore solution of composite cements compared to plain Portland cement due to reduction in clinker content; except for SCMs inherently loaded with alkalis in them. Calcined clay with over 40% kaolinite is often known to consume portlandite completely by 28 days and reduce the pH [20]. Additionally, the use of calcined clay in combination with carbonate source to attain high-volume replacement can also lower the pH further due to reduced clinker content. Chloride binding in cement hydrates increases with this pH reduction [129–131], and this was found to be true in calcined clays binders as well [103]. However, the increased chloride binding upon slight reduction in the pH was explained by combinations of factors such as increased binding by AFm and difference in amount and composition of C–A–S–H phases [105, 129, 132, 133]. Other studies have reported that this effect of pH on the chloride binding by C–(A)–S–H is more prominent in pH of range of 10–12 and often requires sufficient Ca^2+^ ions to be available in the pore solution for their adsorption on the negatively charged C–(A–)S–H surface and the formation of a Stern layer [118, 129]. Increasing the Ca^2+^ concentration in the pore solution (e.g., due to exposure to CaCl_2_) has been shown to increase the chloride binding capacity of a system significantly [99, 134]. Next to the effect on the physical chloride binding, the Ca^2+^ concentration in the pore solution also affects the chemical chloride binding by AFm phases especially in Al-rich systems, such as composite cements containing calcined clays [99]. If sufficient amounts of Ca^2+^ ions are available in the pore solution, they can react with alumina delivered from the calcined clay to form additional AFm phases, which can bind chlorides [95, 99, 134–136].

**Fig. 3:**
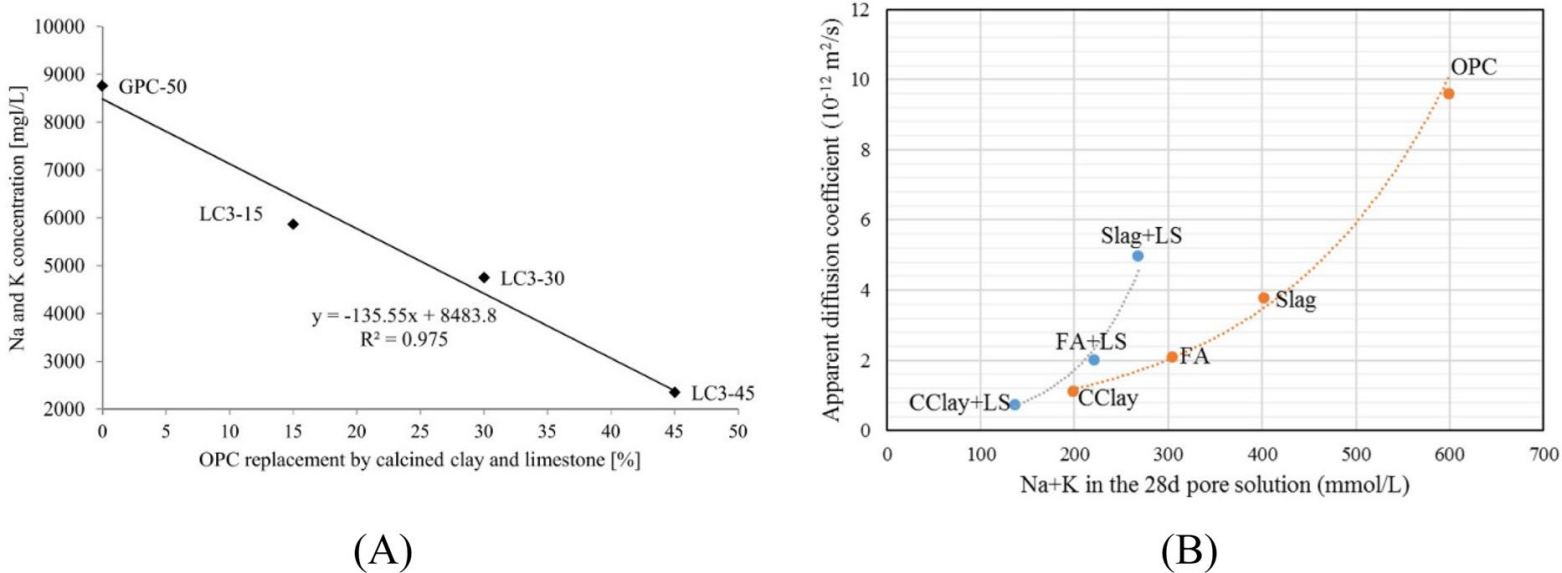
**A** Reduction in total alkalis content in the pore solution with increasing replacement of calcined clay-limestone from [141] and **B** Relationship between total alkalis content vs. apparent diffusion coefficient from [100]. Reprinted from [100] with copyright permission from Elsevier

The changes in the pore solution composition also influence the conductivity of pore solution, which governs the diffusion of ions in the microstructure of cementitious systems that contains interlinked capillary porosity that are saturated with pore solution [137–140]. Diffusivity of ions in the solute of a permeable medium has often been linked to conductivity of the solute using the Nernst-Einstein relationship [140]. Figure 3A presents the reduction of total alkali content in the pore solution with increased replacement level of calcined clay and limestone combinations. The Na + K content decreased by nearly 70% for 50% replacement of the clinker content [141]. In [100], The variation of alkali contents in the pore solution was correlated with the variation of chloride diffusion coefficients in fly ash, slag and calcined clay blended cement systems and the results are reproduced in Fig. 3B from [100]. This shows that lower diffusion coefficient can be obtained for cementitious systems with lower pore solution conductivity such as LC3 systems, despite an equivalent porous network compared to other blended cement pastes [100].

### Physical aspects determining chloride ingress

**Fig. 4:**
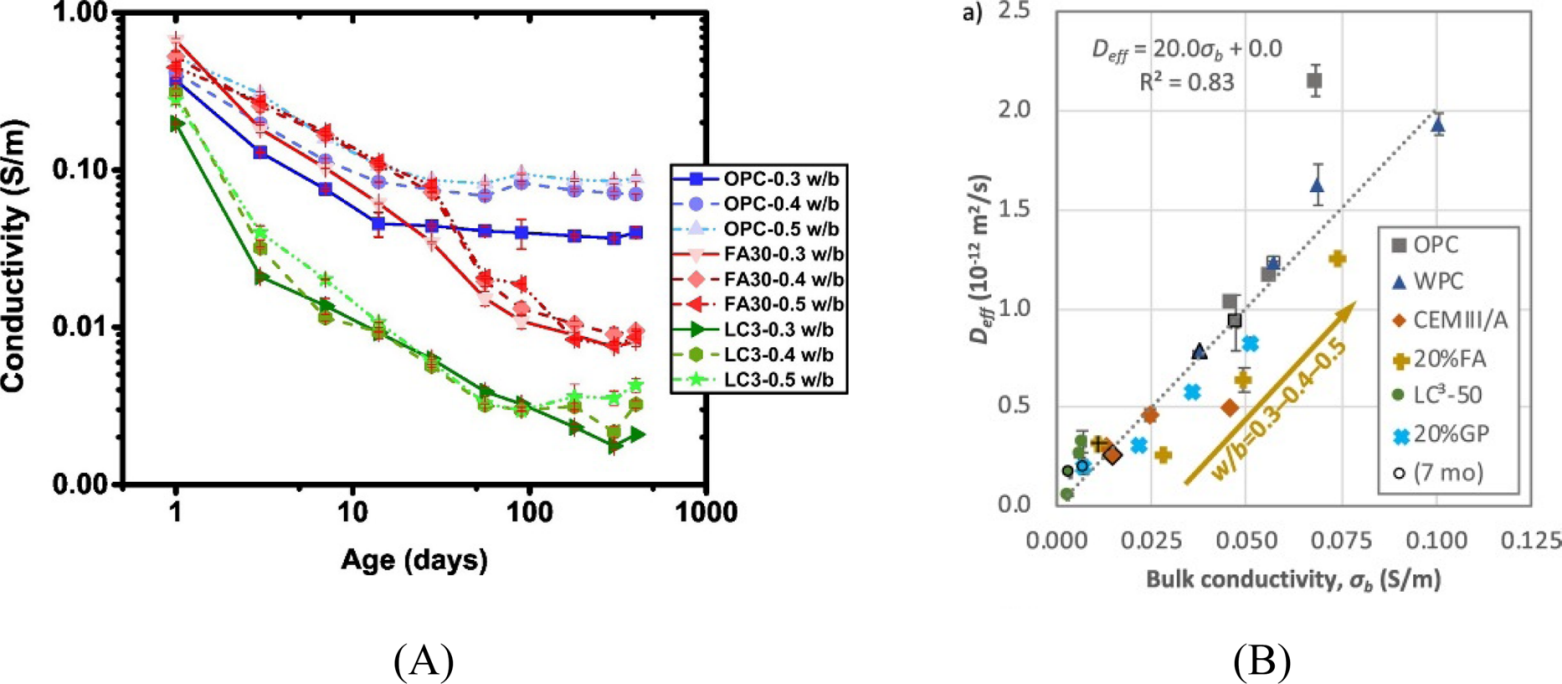
**A** Comparative drop in conductivities for fly ash and calcined clays with varying water-binder ratios from [144] and **B** correlation between bulk conductivity and effective chloride diffusion coefficient [147]. Reprinted from [144, 147] with copyright permission from Elsevier

Deleterious substances, such as chloride ions, are transported into cementitious materials via a combination of different physio-chemical processes, such as diffusion, absorption or convection [142, 143]. Ionic transport in well-cured composite cements is generally slower/reduced compared to plain Portland cement [99, 101, 104, 144–146]. A systematic assessment of factors affecting the chloride diffusivity has shown that the reduction in chloride diffusivity observed in systems containing calcined clay is due to a combination of various factors [147]. The overall transport property of water, gas and ions was often described to be significantly dependent on the total porosity and pore structure of cement matrix [8, 148–150]. Calcined clays additions do not necessarily decrease the total porosity of the cement paste matrix [20, 44], rather resulting in significant refinement of pore size and connectivity [20, 144, 151]. Generally, binders containing calcined clays or a combination of calcined clays and limestone as SCMs are shown to exhibit an excellent resistance against chloride transport [99, 101, 104, 144–146]. Hardened cementitious materials comprise pores of different sizes, which are connected randomly, creating complex paths including impermeable pores and tortuosity for ion diffusion. Apart from the total porosity, the critical pore diameter obtained by MIP, as an indicator for the pore size distribution, exhibited linear correlations with chloride ion diffusion coefficients as reported by several studies [149, 152]. The amount and the connectivity of capillary pores are considered the most important physical parameters controlling the diffusivity of chloride ions because a large part of water and ionic transport occurs through the larger pores (capillary pores) of the microstructure [10, 149]. The (pozzolanic) reaction of SCMs leads to a refinement of the porosity and an increase in gel pores, which reduces the connectivity of the porosity. This is beneficial in reducing water and ionic transport in concrete, resulting in the good performance of binders containing SCMs such as calcined clays [10, 44, 144, 149, 152]. Calcined clay, in particular, is known to cause significant refinement of pore sizes at early curing ages [20, 44]. However, this effect does vary with the kaolinite content of the clays. Increasing kaolinite from 20 to 50% was found to cause significant pore refinement by 7 days and any increase in kaolinite content had negligible impact on the critical pore entry diameter [20]. In line with the trends of pore refinement with kaolinite content, the diffusion coefficient was found to reduce with increase in kaolinite content from 20 to 50% and remained nearly similar with further increase in kaolinite content [104]. Binary and ternary binders containing limestone were studied for chloride ingress using diffusion experiments using 4 clays with varying kaolinite content from 20 to 65% in [153] and the chloride penetration depth reduced with increasing kaolinite content, confirming the positive influence of kaolinite content and corresponding pore refinement. In order to compare the evolution of pore structure with other SCMs, calcined clay cements were monitored using conductivity measurement on cement paste for early pore refinement [44, 144] and the results are presented in Fig. 4A. Calcined clay was found to cause a significant drop in conductivity by 3 days, correlating well with pore refinement observed using MIP in [20, 44]. Since conductivities are often linked to chloride diffusivities, such relationships were found to be valid for calcined clay concrete as well, which is in line with other SCMs. Figure 4B shows the relationship between bulk conductivity and effective diffusion coefficient showcasing that bulk conductivity could be used to explain chloride ingress in most SCM blended cementitious systems, including calcined clays.

Though the features of capillary pores control the ionic diffusion including chloride, the complex physio-chemical phenomena induced by surface charges in smaller pores i.e. gel pores [116, 117, 126, 154–159] cannot be overlooked when describing the transport of chloride ions in concrete. Also, since the physical properties govern the ionic transport in hydrated cementitious materials, it is important to mention that the pore structure of cement paste is different from mortar and further extending to concrete due to the presence of interfacial transition zone (ITZ) formed with the presence of aggregates [129, 160, 161]. The pore structure in the vicinity of ITZ can also influence the transport properties [162–167]. This highlights the importance of sampling for the establishment of any correlation between pore size distribution results and concrete performance. In addition to increased porosity in ITZ, aggregates can introduce variation to the total porous medium i.e., hydrated cementitious matrix in the system. Some studies have attempted to capture in the change in physical structure in concrete using tortuosity factors. Tortuosity is a measure of the distorted path in the pore network [168]. Increased tortuosity was reported to decrease the chloride diffusion coefficient as well as oxygen diffusion [144, 147, 169]. Increased tortuosity factors were used to explain the significantly improved chloride resistance of calcined clay concrete compared with other SCM types [151, 170]. However, these concepts have not been fully explored to allow a quantitative understanding of these effects on ionic transport in microstructures.

## Chloride ingress in concretes containing calcined clay

### Applicability of existing testing methodologies for chloride ingress to concrete containing calcined clays

**Fig. 5:**
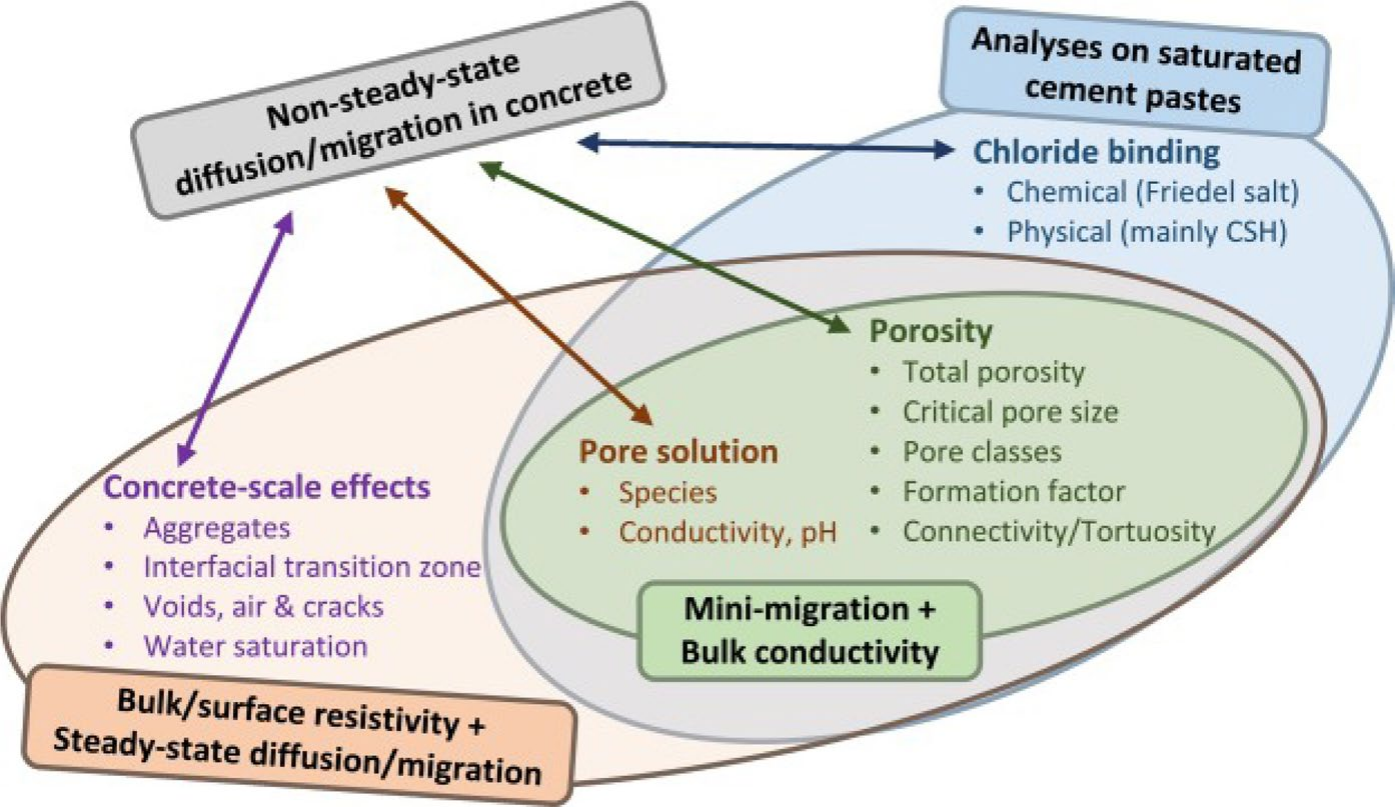
Parameters included in different types of experimental tests, as reported in [147]. Reprinted from [147] with copyright permission from Elsevier

Several direct and indicative test methods are used to evaluate the chloride resistance of concrete mixes with the aim of assessing the performance of new SCMs or combinations of SCMs and for specifying performance criteria for concrete to be used in such aggressive environments [143, 149, 171–180]. Often some of the testing methods, specifically accelerated testing methods, capture only a certain portion of contribution that are linked to performance in chloride exposure. For example, the factor governing the non-steady state diffusion/migration coefficient schematically was classified in [147] and the schematic is reproduced in Fig. 5 to highlight various parameters influencing the chloride transport parameter. While factors related to scales of paste, such as binding, pore structure, and pore solution are captured in studies done on paste/mortar in literature, as discussed in Sect. 2, the concrete scale effects, such as, aggregate size and volume, interfacial transition zone (ITZ), distribution of voids (like air entrainment) are often neglected in studies carried out in paste/mortar [152, 164, 181, 182]. As the construction industry is keen to transition towards performance-based specifications, it is important to ascertain the ‘true performance’ in the scale of concrete and develop limiting values for specifications that are scientifically sound to ensure desired performance. Various standardised testing methods available to determine the performance at the scale of concrete are summarised in Table 1, from [183]. These testing methods are widely used in performance specifications to evaluate the quality of concrete including calcined clay-based concrete. Remarks on the use of these testing methods for calcined clay concrete based on published data are also provided in Table 1. Some of these factors are discussed in the following sections in more detail.

**Table 1:** Testing methodologies for chloride ingress and its usefulness for calcined clay concretes based on [183] with remarks on suitability for calcined clay based concrete

Name of test method	Standards	Parameter obtained	Remarks	Remarks on calcined clay systems	References
Chloride diffusion test	ASTM C1556 [172, 173]	Diffusion coefficient	More realistic but challenging to perform in terms of handling samples and chloride content measurement across different depths. A minimum of 35 days of exposure and more time required for high-performance concrete	Longer exposure time maybe required for calcined clay to obtain sufficient depth of chloride ingress due highly refined pore structure. Typically, ingress depths of 5 mm are observed. However, the surface effect may also influence in the initial depth. Extended exposure beyond 35 days is recommended for calcined clay concrete	[100, 104, 146, 153, 170, 195, 196]
Rapid Chloride migration test	NT Buld 492 [174]	Non-steady state migration coefficient	Relatively quick to perform with testing duration is 2–4 days. It is estimated based on the depth of penetration of chloride, which is more appropriate than current-based measurement—no heating of specimen due to reduced voltage for lower quality concrete	Migration coefficient have been related to the diffusion coefficient for calcined clay concrete. Due to very high initial resistivity, the required Voltage is about 60 V for calcined clay concretes and duration of 96 h is required for calcined clay concrete Lack of relationship with long-term diffusion data for calcined clay concrete may be limiting the usage and conversion factors for migration to diffusion needs to be developed for calcined clay concrete for use in service life design	[128, 144, 145, 147, 183, 189, 197]
Rapid chloride permeability test	ASTM C 1202 [175]	Total Charge passed	Easy to perform. Popular in the construction industry. Testing duration: 6 h + 1 day of specimen conditioning. Sensitive to specimens heating which could affect the measurement in the case of poorer concretes	Charge passed values for calcined clay concrete are found to be significantly low, often below 200 Coulombs for calcined clay concrete. Higher resistivity of concrete could favour the ASTM concrete quality guidelines towards calcined clay concrete. Care should be taken while making meaningful comparison of the data obtained	[44, 145, 187]
Bulk resistivity/conductivity/surface resistivity chloride conductivity	ASTM C1876 [176–178]	Resistivity, Conductivity	Time required < 5 min without sample conditioning. Two standards are available in ASTM for measurement based on AC or DC measurement input. Sensitive to the saturation level of concrete and conditioning. Consistent results can be obtained with proper saturation	Significantly higher resistivity value noted for calcined clay concrete. Different forms of resistivity measurement like bulk and surface are available which provide different values. Interference from pore solution and saturation level can affect the data. Testing protocol should be clearly stated in instance of using this measurement	[44, 82, 83, 100, 144, 145, 151, 183, 187, 198–202]
	AASHTO T 358 [179]	Surface resistivity	time required < 5 min without sample conditioning. Essential to maintain specimens properly saturated. Measurement can be quite easily adapted for onsite measurement for assessing the quality of cover concrete		
Formation factor	ASTM C1876 [176, 180]	Formation factor	The conductivity of the pore solution is required. Pore solution conductivity can be tedious to obtain	Useful to avoid interference from lower pore solution alkalinity of calcined clay concrete. Although pore solution is integral part of explaining chloride ingress, deviation from in pore solution between cement types, concrete ingredients and pore solution composition in actual field condition can interfere with the conductivity measurement which can avoided using this approach	[144, 147, 187, 199]

The bulk diffusion test (ASTM C1556, EN 12390-11 or NordTest NT BUILD 443-chloride diffusion test) remains the closest to natural chloride exposure involving direct immersion in chloride-rich solution, followed by profiling the total chloride content in the sample as function of the depth from the exposed surface. The test can be laborious and time-consuming due to rigorous sample collection and testing on chloride content in multiple batches of concrete powder samples. In the ASTM and NordTest methods, the concentration used in laboratory diffusion experiments is about 2.8 M (i.e., 165 g/l) instead of 30 g/l NaCl concentration that is typical of seawater (and which is used in EN 12390-11) to ensure accelerated conditions and also assessment related to de-icing salts conditions.

Other forms of rapid testing methods are typically based on driving the chloride into concrete using an externally applied electrical potential, similar to the works of Tang and Nilsson from 1990s [171]. Such accelerated test methods include ASTM C1202 [175], NT BUILD 492 [174], and other variants of migration test [147, 171, 184]) are often preferred. Although these test methods provide a rapid and acceptable levels of performance assessment, these methods are highly criticised for applicability to different blended Portland cements and alternative cement types. Accelerated chloride migration test is referred in the Model Code for service life design (FIB 34) [185]. However, other rapid test methods like ASTM C1202, ASTM C1876, AASHTO T 358 etc. are constantly studied for extending their validity for new SCMs or combination of SCMs like calcined clay, calcined clay-limestone combinations [149]. These studies are crucial to ensure the uptake of newer breeds of SCM types. Several recent studies have attempted to establish such relationships between accelerated chloride resistance test and diffusion coefficient for calcined clay and calcined clay-limestone systems [82, 83, 128, 144, 147, 183, 186–194]. A recent paper investigated the suitability of these methods to test LC3 concrete [183, 195, 196] by assessing the performance of each accelerated testing method and comparing the results using ASTM C1556 bulk chloride diffusion test as reference. Results obtained using ASTM C1556 bulk diffusion test showed that, the resistance of LC3 concrete against chloride diffusion was greatly improved compared to that of reference OPC (referred to as GP, general purpose cement in Australia context) cement-based concrete. The apparent chloride diffusion coefficients of LC3 concrete was 4–5 times lower than that of reference Portland cement-based concrete [99, 104, 144, 147, 192]. However, there is still limited published long-term data on realistic exposure conditions, specifically with low purity clay which is gaining attention in the recent years.

### Transport properties and chloride resistance of concretes containing calcined clays

#### Impact of kaolinite content, curing duration and time-dependency on performance

**Fig. 6:**
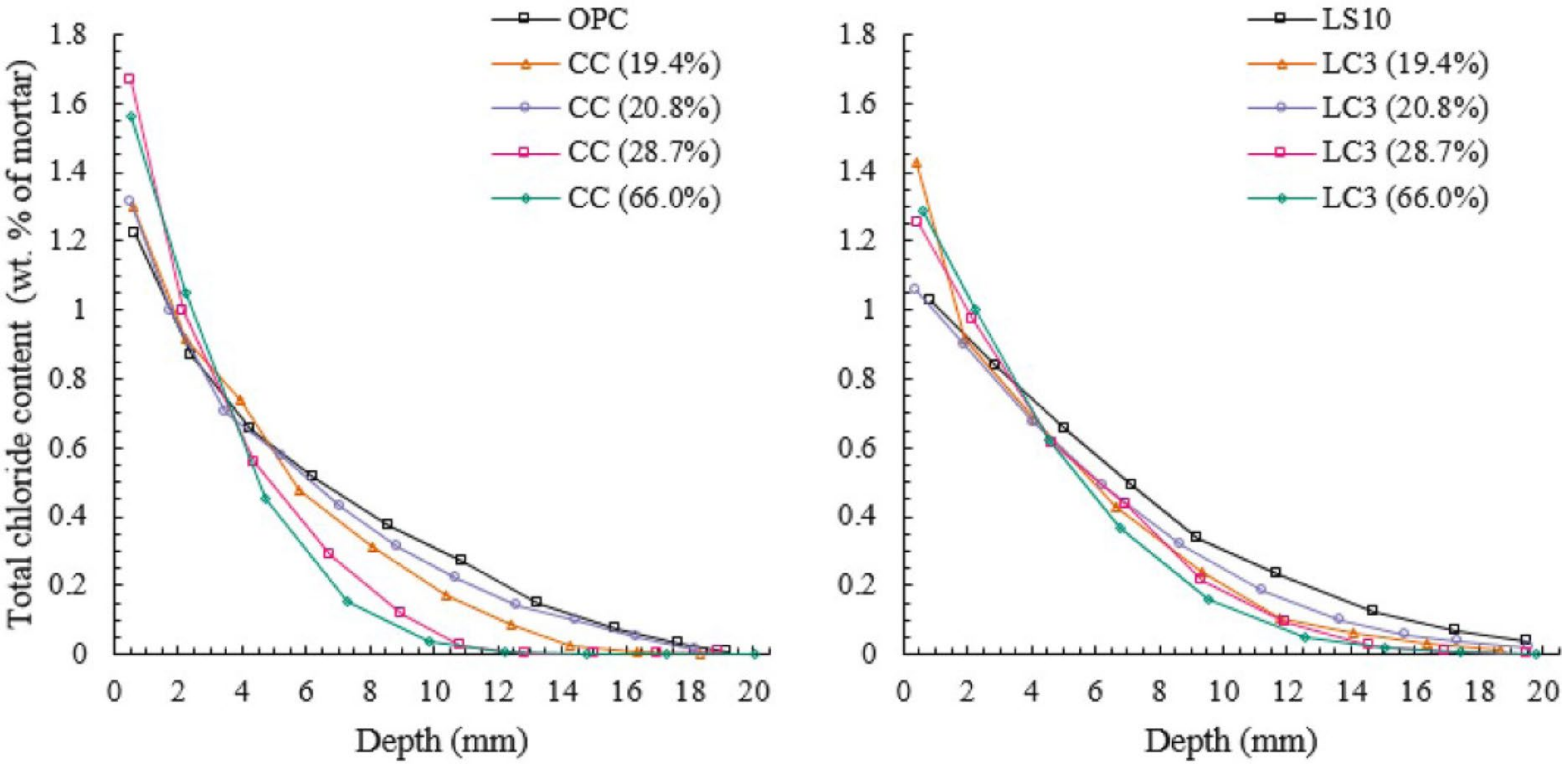
Influence of kaolinite content on chloride ingress in calcined clay (CC) and calcined clay-limestone (LC3) mortars, from [153]. Reprinted from [153] with copyright permission from Elsevier

SCMs are widely recognised to positively influence the pore structure over time [44, 203–207] and hence, improving transport properties and resistance to chloride ion penetration over time [208–212]. Some of the factors involved in the time dependent changes are curing duration, reactivity of the SCM, water-binder ratio, replacement level etc. [213, 214]. For calcined clay concrete, kaolinite content is found to significantly influence the chloride ingress and chloride diffusion coefficient [104, 153]. Results on binary (labelled CC) and ternary LC3 cement mortars prepared with four different kaolinite contents are presented in Fig. 6, it is seen from the data that chloride ingress is limited with an increase in kaolinite content from 20 to 65% [153]. Similar reduction in diffusion coefficient with kaolinite content up to 50% was reported in [104]. Thereafter the diffusion coefficient remained similar with no further reduction, highlighting that further increase in purity doesn’t necessarily improve performance and that low to moderate purity of about 50% is sufficient to produce an improved performance against chlorides.

**Fig. 7:**
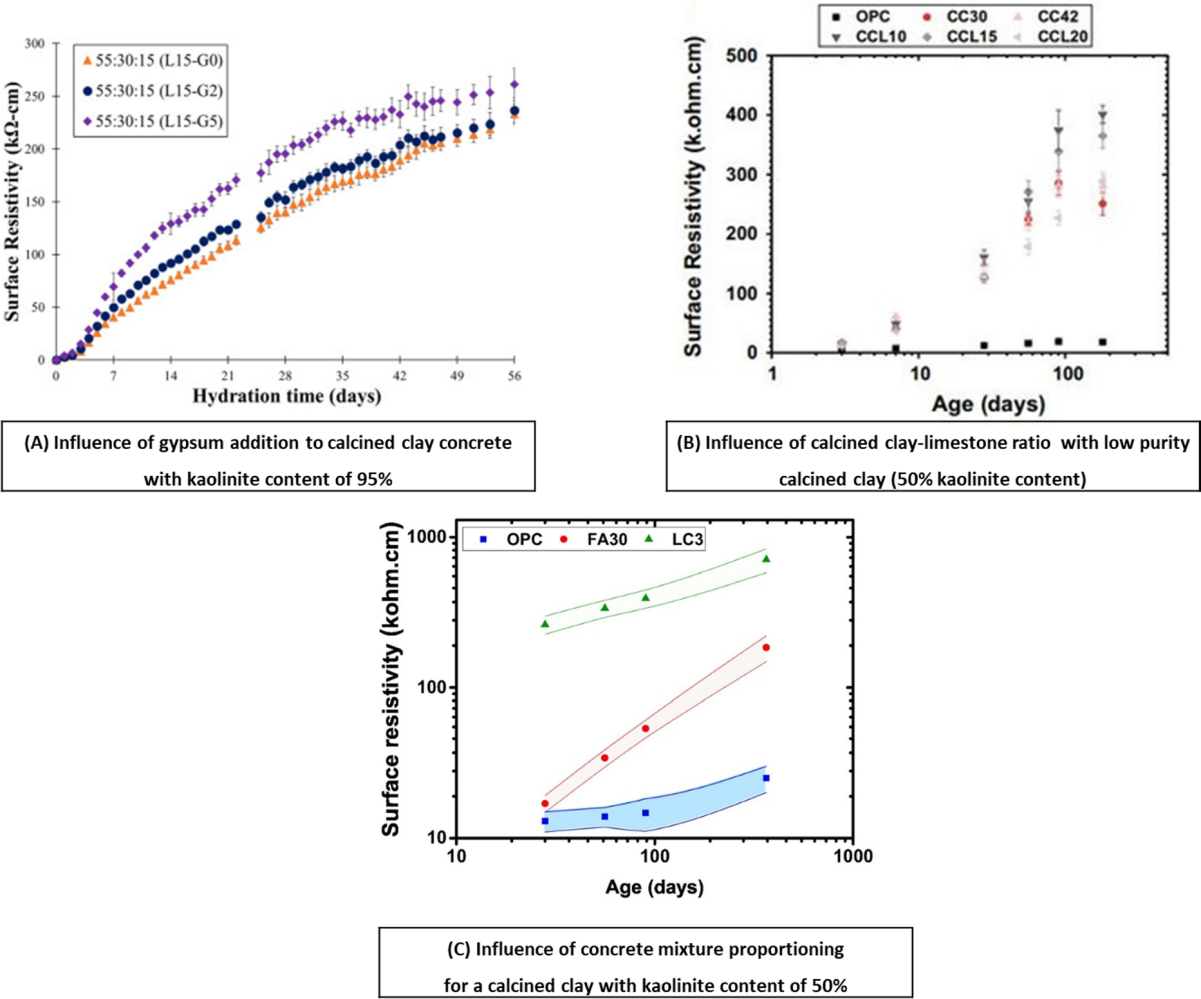
Surface resistivity development in calcined clay binder. **A** Influence of gypsum addition from [199], **B** influence of calcined clay-limestone ratio from [202], and **C** influence of concrete mixture proportioning from [144]. Reprinted from [144, 199, 202], with copyright permission from Elsevier and Springer

In case of low reactivity SCMs like fly ashes, the reactions may continue for a longer period of time, thereby resulting in a significant improvement at later ages and make it crucial to incorporate the curing duration and/or time dependency in estimating the long term performance [204, 206, 207]. While calcined clays made of kaolinitic clays are known to rapidly develop refined pore structure, low purity with lower level of kaolinite content or less reactive alternative clay minerals might require extended curing to positively influence the performance. Since chloride exposure predominantly occurs in saturated or high humidity conditions, concrete microstructure can continue to evolve in such conditions. Also chloride ions typically do not manifest in terms of physical damage, at least for sodium chloride, allowing continuous densification of pore structure; thereby reducing the ingress rate over time [215, 216]. Resistivity of concrete is a simple measurement to monitor the time-dependency of transport properties in concrete and it is found to correlate well with other transport properties [44, 138, 217–222] and chloride diffusion, as highlighted in Sect. 2.2. Resistivity development in calcined clay-based concrete mixes can be influenced by the composition, blend design and calcined clay-limestone ratio. For example, results from [199] about additions of gypsum for sulphate balance in calcined clay, as presented in Fig. 7A. The results indicate that gypsum addition can modify the resistivity development with mixes containing 5% gypsum showing higher resistivity throughout the curing duration. Dhandapani and Santhanam [202] studied various calcined clay-limestone ratios for 50% kaolinite content clays and found that calcined clay-limestone combination containing 10–15% limestone showed synergistic resistivity development compared to binary calcined clay (both at 30 and 45% replacement), as shown in Fig. 7B. Another common observation is that resistivity development in concrete is significantly higher for calcined clay at early age, irrespective of mix design and water-binder ratio compared to other common SCMs, such as fly ash, slag etc. This was confirmed from resistivity monitoring on calcined clay and fly ash concrete on three set of concrete mix designs as shown in Fig. 7C, reproduced from [144]. This has been linked to early refinement of pore structure as discussed in Sect. 2.2. Higher resistivity at early ages implies that transport properties are considerably reduced at early ages and significantly lower chloride diffusion coefficient can be attained with calcined clay containing as low as 30–50% kaolinite content even with shorter curing duration [187].

Time-dependent change in the chloride transport parameter is caused by progressive refinement of the pore structure and the reduced flux of free chloride for further diffusion due to chemical binding. These factors affect the diffusion coefficient obtained from the chloride profile over extended exposure time. Studies have attempted to mathematically denote the time dependency using Eq. 1. Time dependency of chloride transport parameters significantly influences the long-term chloride penetration estimation [223–226]. The simplest form of the long-term chloride prediction is based on Crank’s error function solution [227] for Fick’s second law, assuming a constant value for chloride diffusion coefficient and surface chloride concentration [185, 224, 225]. This involves the use of a time dependency function to account for the reduction of chloride diffusion with time using the fitting constant known as the decay coefficient or ageing factor [225]. The ageing factor can be obtained using the negative slope of diffusion coefficient with time plotted on a log–log scale [226, 228–230].

1 $${{\text{D}}_{\text{cl},\text{ t}} = {\text{D}}_{\text{cl},\text{ ref}}\left(\frac{{t}_{ref}}{t}\right)}^{m}$$

where

D_cl, t_ = Diffusion coefficient at exposure time t,

D_cl, ref_ = Diffusion coefficient at a reference time t_ref_,

m = Ageing or decay factor.

A higher value of the ageing coefficient indicates the continuous reduction in diffusion coefficient due to pore refinement or reduced flux from chloride binding. Both these factors can lead to a higher decrease in the diffusion coefficient over time. The ageing factor for OPC concrete and the common SCMs based concrete, like slag (i.e., ground granulated blast furnace slag, GGBS) and fly ashes, are specified in FIB model code (as shown in Table 2) [185], while recommendations are not available for calcined clay concrete. The available data from the literature is summarised in Table 2. A concrete made with Ordinary Portland Cement (or CEM I) usually exhibits a lower level of decay with the ageing factor varying from 0.2 to 0.3 whereas concrete with blended cement can have a higher decay ranging from 0.5 to 0.7 [223]. Although the FIB model code specifies certain decay value for a particular mineral admixture, other factors, such as reactivity, replacement levels, water-binder ratio may influence the value. No systematic studies on time dependency with kaolinite content or other low purity clays are available in literature, warranting more studies on this aspect in future. As more and more multi-component composite cement types are being standardised, robust methods or recommended values with suitable safety factor to obtain such input parameter to model long-term performance needs to be developed as combinations of two or more SCM can again lead to variability in ageing coefficient. For example, no distinction is made for the composite cement based combination of SCM or with/without limestone for recommendation made in FIB model code. On a parallel development, more sophisticated modelling methods based on fundamental principles on diffusion laws accounting for materials reactivities are being developed [231] which could enable to model and predict performance of composite multi-component cement. However, this modelling is yet to be deployed widely in engineering communities for concrete construction.

**Table 2:** Comparison of ageing factors from FIB and literature

Ageing factor from FIB34		Ageing factor from literature		References
Binder type	m	Binder type	m	
OPC	0.30	OPC	< 0.40	[224, 226, 228, 232]
Fly ash	0.60	fly ash	0.40–0.80	
Slag	0.45	slag	0.40–0.60	
Metakaolin (< 20% replacement)/calcined clay (< 40% replacement)	NA	Metakaolin (< 20% replacement)/calcined clay (< 40% replacement)	0.3–0.6	[144, 228, 233]

#### Impact of concrete mixture proportioning

**Fig. 8:**
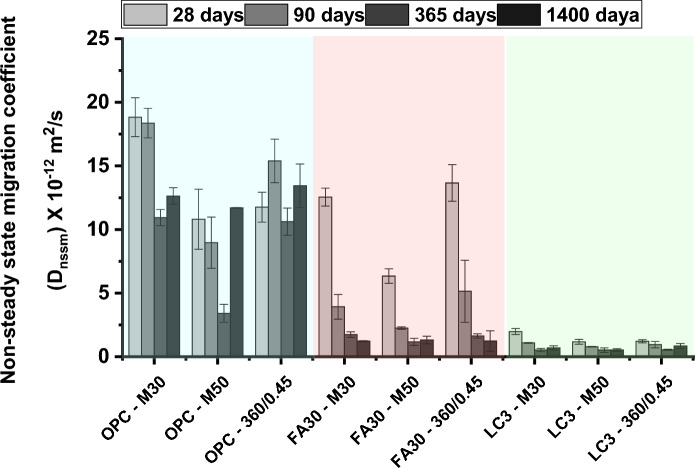
Chloride resistance of different strength grade of concrete on the long-term chloride resistance of LC3 concretes containing calcined clay with 50% kaolinite content, adapted based on data presented in [144]

Modifying the concrete mixture proportioning, in terms of binder content and water-binder ratio, is widely practiced to control strength requirement and chloride resistance of concrete mixes with available source of SCMs. For example, in the United Kingdom, BS 8500 [234] recommends varying binder content and water-binder ratio for different cement types for prescriptively specifying concrete in different exposure classes. Modifying the mixture proportioning can also be done to attain levels of resistance as prescribed in the concrete performance specification during mix development phase of the construction project. Studies dealing with materials characteristics such as kaolinite content in clay or calcined clay-limestone ratio are often carried out on mortars or paste which is reasonable for scientific evaluation, but, doesn’t necessarily provide the limiting values meeting concrete specification based on combined strength and durability criteria. In a study reported in [145], the non-steady state migration coefficient in calcined clay concrete made with three mix designs was monitored for over 4 years (1 year in curing and remaining 3 years natural exposure). Two concrete mixes were tailored to produce M30 and M50 grade concrete (with normal Portland cement, 30% fly ash blend, and LC3) and the third mix was produced with similar mix design (360 kg/m^3^ binder content and 0.45 w/b). The results are replotted from the original work reported in [145] and presented in Fig. 8. It was found that calcined clay (50% kaolinite content) concrete showed 10 times lower migration coefficient compared to OPC concretes and this difference remained valid in the long term for a period of 1400 days. The study also compared calcined clay-limestone concrete with three fly ash concrete that managed to show improved performance over time from 90 to 1400 days. Similarly, calcined clay with 50% kaolinite content was found produce 5 times lower diffusion coefficient in concretes in the study reported in [195].

For alternative clay types, chloride profiles of kaolinite clay mixes were compared with illitic clays, and the results showed that kaolinite clay marginally lowered the chloride content across the depth compared to the illitic clays mixes [46]. However, concrete containing other low purity clays and alternative clay types are not well reported for their long-term chloride resistance performance. Based on information available in literature, clays with kaolinite content of about 50% can provide significant improvement in chloride resistance of the order of 5–10 times lower compared to OPC concrete across range of mix designs which shows it is possible to significantly improve the chloride resistance across a wide range of concrete strength without necessarily increasing cement content to improve the durability performance [59, 100, 104, 144–147, 151, 153, 235, 236]. Recent studies on the use of LC2 showed that calcined clay concretes produce similar early improvements in resistivity across several concrete mixes compared to fly ash concrete. Most of the improvements with calcined clays occurred between 3–7 days, as compared to fly ashes that took more than 28 days to show a positive impact, specifically at higher water-binder ratios. Combined use of SCMs with different levels of reactivities for improving concrete performance during concrete mix design stage can be a potential route for uptake of calcined clay along with other SCMs. There is also possibilities of using a combination of clays with varying reactivities or varying calcined clay-limestone ratios to significantly reduce CO_2_ footprint of the concrete and ensuring performance criteria are suitably met based on the demands of the exposure conditions, as shown in literature for LC3 concrete [190, 237] and LC2 additions in concrete [82].

#### Comparison of calcined clay performance with other SCMs

Blended cement containing slag and fly ashes are already widely used in aggressive marine environments. Hence, it is practically relevant to compare the performance calcined clay with other common SCMs, such as fly ashes and slags, that are widely utilized for concreting in aggressive environments. On comparing metakaolin to slag (GGBS based) concrete with respect to chloride conductivity index, 20% metakaolin was found to show better performance compared to 50% GGCS concrete at all w/b ratios. Similarly, concretes containing low purity calcined clay were found to perform significantly better than fly ash concrete at early ages while fly ashes were found to require extended curing or reduced w/b for improving the performance [145, 187].

**Fig. 9:**
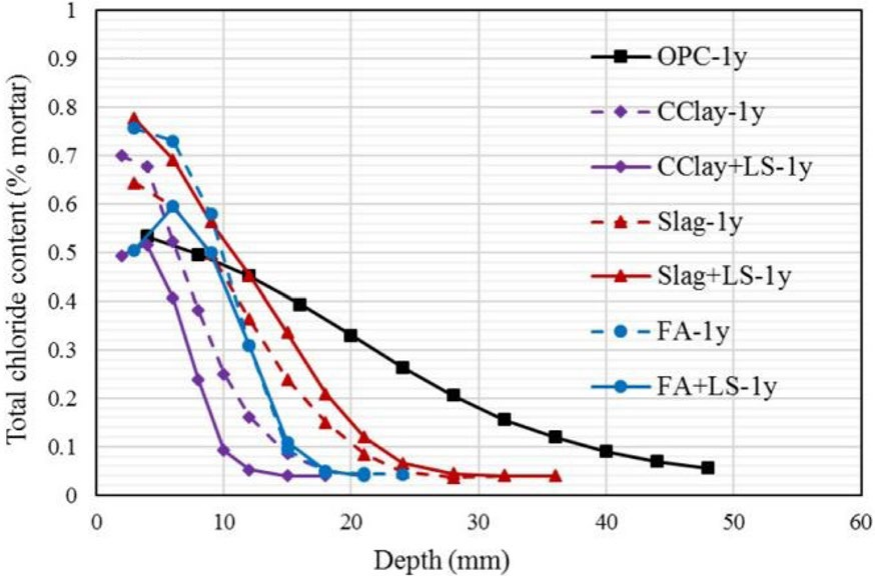
Chloride profile of calcined clay in comparison with other SCMs determined after 1 year (1y) of exposure [100]. Reprinted from [100] with copyright permission from Elsevier

The chloride profile of binary and ternary blended systems (with calcined clay) after 1 year exposure of 0.5 M NaCl solution [100] is shown in Fig. 9. OPC exhibits largest chloride penetration depth while calcined clays binary (CClay in Fig. 9) and ternary (CClay + LS in Fig. 9) systems shows better resistance to chloride penetration in comparison to other SCMs (fly ash and slag with/without limestone). The chloride penetration depth of systems containing calcined clays and limestone is similar or smaller compared to binary systems containing only calcined clays as SCMs [100]. Other studies, also demonstrated superior chloride resistance of calcined clay compared to other SCMs [82, 100, 144, 189, 191, 202, 238].

#### Chloride ingress in carbonated concrete

**Fig. 10:**
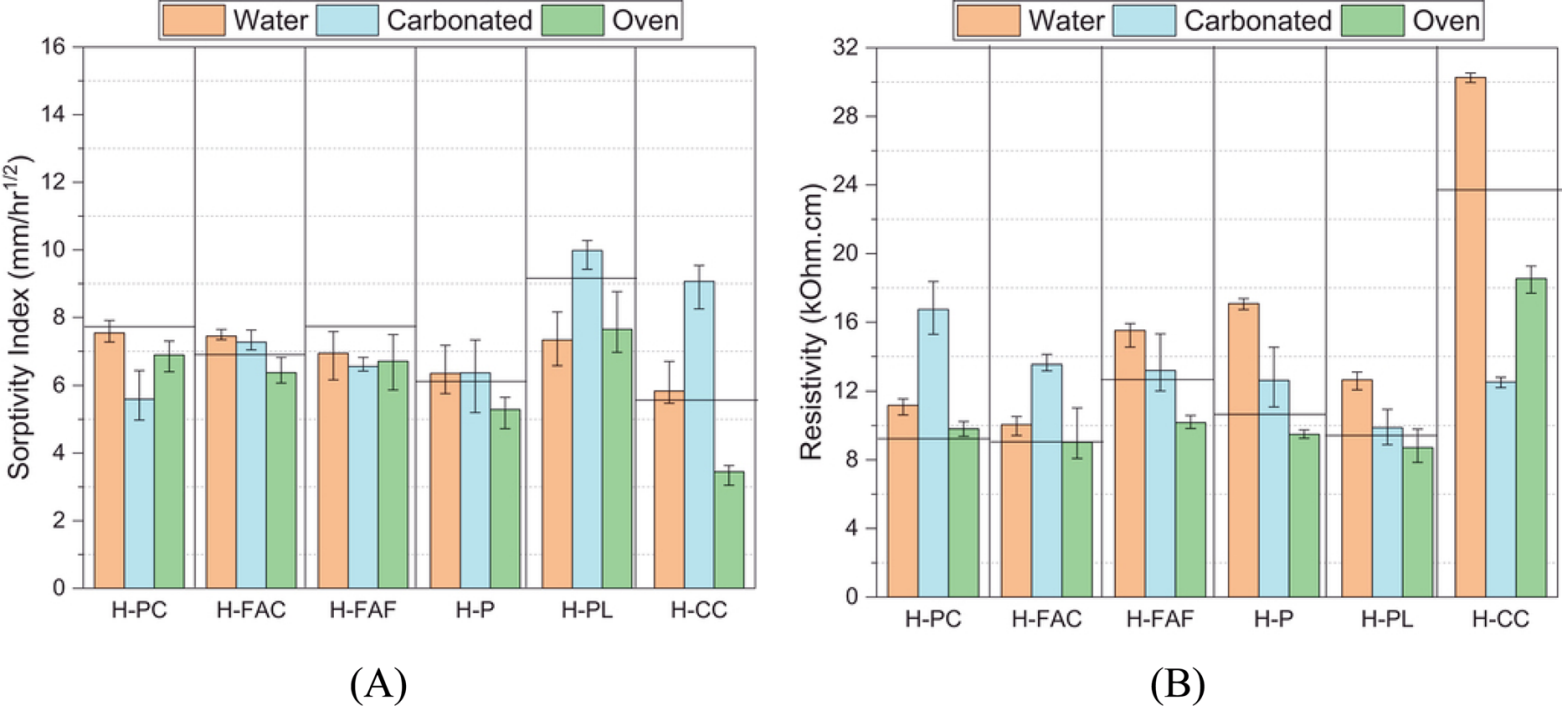
Transport properties in carbonated concrete [240]. Reprinted from [240] with copyright permission from Taylor & Francis

One of the key factors to consider for long-term modelling of chloride ingress in low clinker cements is the effect of surface carbonation, which can be a likely scenario in the long term, at least in some instances of air-borne chloride exposure. High volume clinker replacement by calcined clay or using calcined clay-limestone blends leads to reduced carbonation performance and this is summarised in the previous review from the TC in detail [37]. This trend is true for all low clinker concretes. However, the possibility of carbonation in dominant chloride exposure region like tidal or submerged conditions can be significantly lower due to the fact that carbonation tendency reduces at higher humidity conditions. Nevertheless, considering the long-term service life, often > 100 years for some critical infrastructure, the possibility of surface carbonation cannot be neglected, specifically in low clinker cements. Hence, it becomes essential to consider the porosity, transport properties and chloride resistance in carbonated concrete, specifically in low clinker cements, that are prone to carbonation leading to significant pore structure alterations [239]. In [240], the change in bulk porosity and resistivity was evaluated in concrete specimens subjected to three conditions i.e., curing, oven drying and carbonation exposure. The results shown in Fig. 10 that transport properties and resistivity of calcined clay concrete (H-CC in the plot) worsened upon carbonation compared to OPC concrete (H-PC in the plot) [240].

**Fig. 11:**
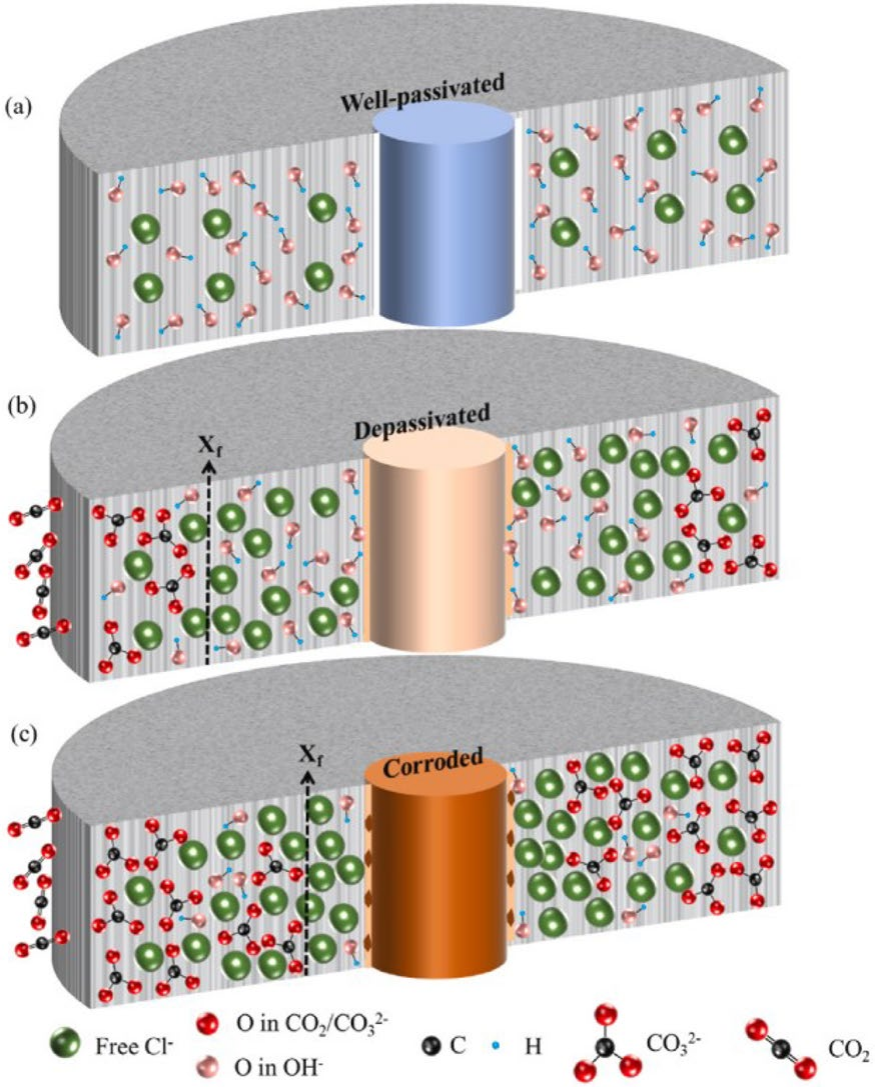
Schematic representation of corrosion mechanism under coupled carbonation and chloride attack, as presented in [242], depicting the release of chloride as free chloride as carbonation front progress towards the reinforcing steel. Reprinted from [242] with copyright permission from Elsevier

Besides changes in transport properties, carbonation of concrete cover could also cause redistribution of the chlorides by increasing the concentration of free chlorides [241]. In [242], the corrosion performance of LC3 paste with pre-mixed chloride upon carbonation exposure was investigated. It was found that the steel reinforcement was protected with lower corrosion rates in the chloride contaminated LC3 [242]. However, carbonation of chloride contaminated concrete was found to significantly reduce the corrosion performance based on observations from the accelerated testing conditions in laboratory. Figure 11 presents a schematic representation depicting the scenario of carbonation of chloride exposed concrete and the possibility of corrosion caused by combined effect of chloride ingress, release of free chloride upon carbonation and reduction of pH due to carbonation. Such scenarios may be crucial in concrete elements which may undergo some levels of carbonation and chloride ingress eventually during the service life with carbonation occurring much more slowly compared to chloride ingress leading to increasing free chlorides. Studies dealing with such combined exposure conditions are quite limited in the literature for low clinker cements and calcined clays concrete, and may require more focus in future, to determine performance in realistic conditions that are possible in field exposure.

## Corrosion resistance and service life prediction of calcined clay concrete

**Fig. 12:**
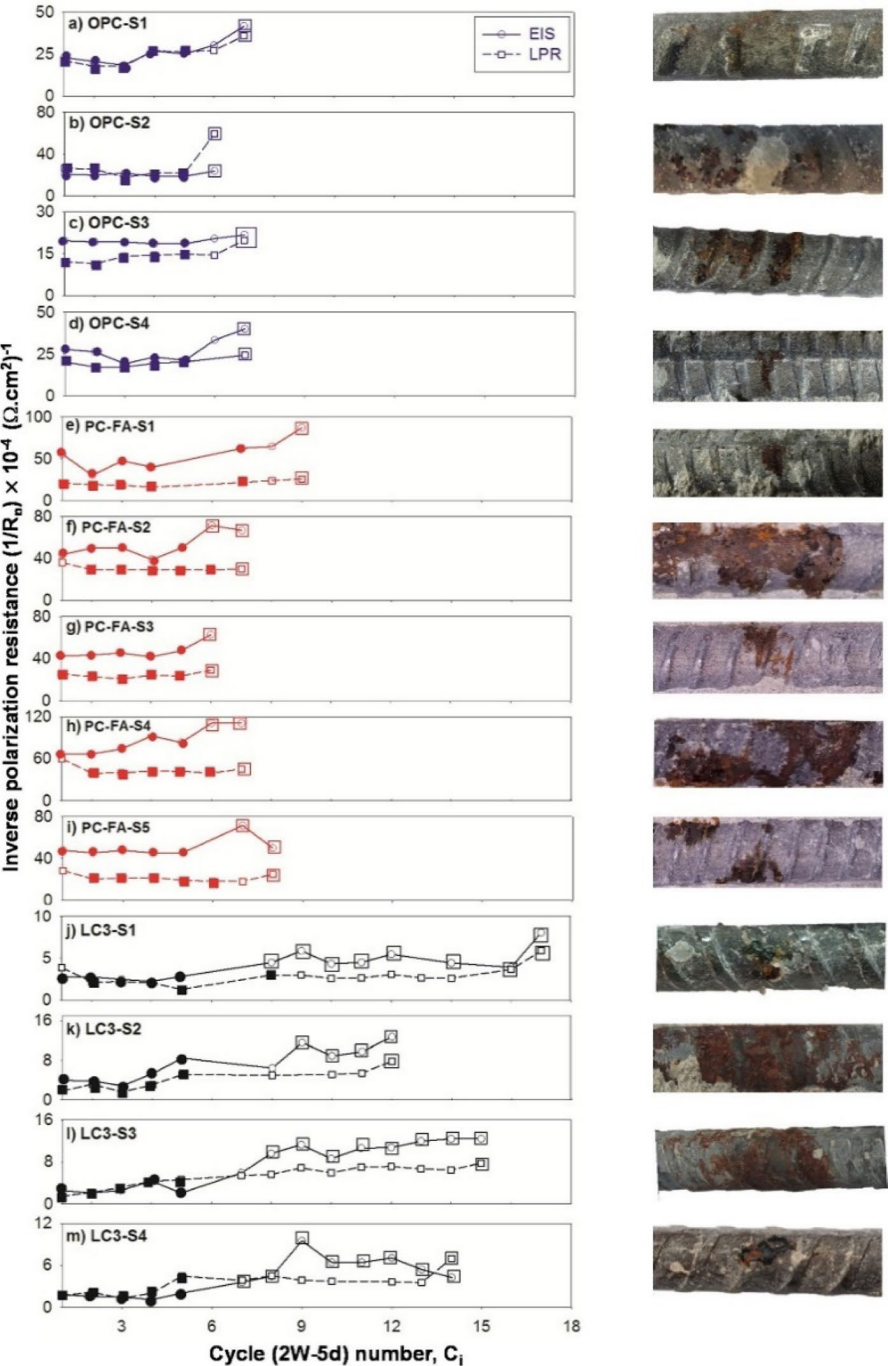
Electrochemical response from EIS and LPR techniques for OPC and LC3 specimens on exposure to chlorides in laboratory conditions [201]. Reprinted from [201] with copyright permission from Elsevier

Reinforcement corrosion in cementitious systems is complex with several factors, including environmental factors like exposure conditions, temperature and relative humidity, properties of cover concrete and steel–concrete interface, influencing the performance [243, 244]. Some of the material parameters that could influence corrosion performance include cement composition and physiochemical properties such as chloride/moisture ingress, chloride threshold, and corrosion rate of steel embedded in cementitious systems. Critical chloride threshold is the chloride content at reinforcement level that is sufficient to produce an acceptable level of corrosion by depassivation [245]. There is a wide range of values reported in literature using different testing methodologies for OPC and blended Portland cements [245, 246]. Due to the high resistivity of concrete containing calcined clay, it is challenging to get an accurate estimate of chloride threshold using existing testing methods [201, 247]. In [201], polarization resistance was monitored to identify the onset of corrosion using linear polarization resistance (LPR) and electrochemical impedance spectroscopy (EIS). The results from this study are reproduced in Fig. 12, and indicate that there is lower chloride build-up at the steel surface due to reduced diffusion coefficient (discussed earlier in Sects. 2, 3). The reflection of this benefit is visible in the results from [201] as well with calcined clay mortars taking nearly 10 additional cycles to show visible signs of corrosion. Despite the longer duration, the critical chloride threshold value observed for the onset of corrosion was almost 50% lower in calcined clay systems (0.2% by weight of cement) compared to plain Portland cement (0.4% by weight of cement). Hence, this needs to be accounted properly during the corrosion assessment and service life design of concrete containing calcined clays. Similar observations on delayed onset of corrosion initiation were reported for metakaolin addition in the range of 10–20% in [248].

**Fig. 13:**
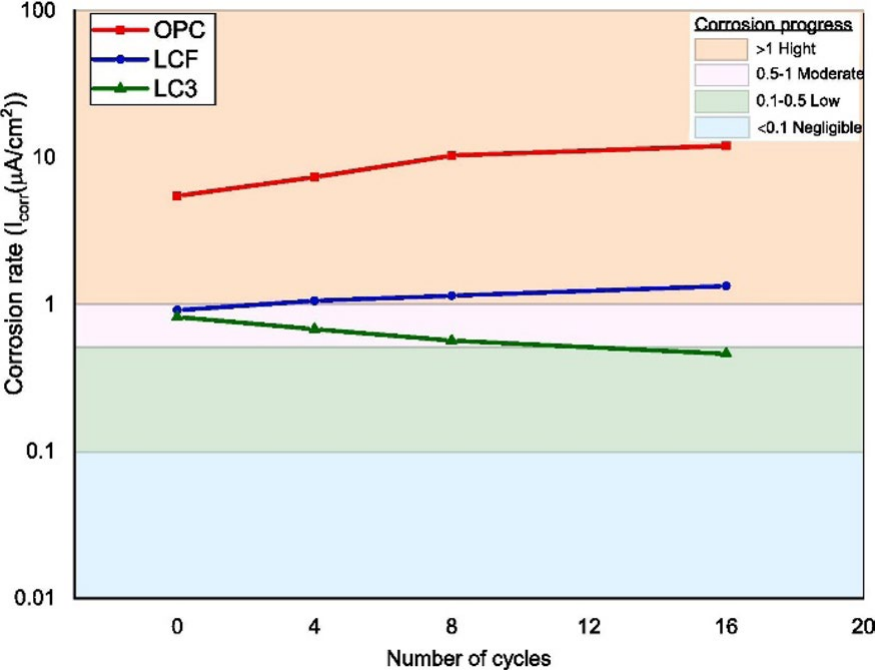
Comparison of corrosion rate in concretes containing between plain Portland cement and calcined clay binders [192]. Reprinted from [192] with copyright permission from Elsevier

Another parameter governing the extent of damage due to corrosion is corrosion rate, indicating the rate at which reinforcing steel corrodes. In recent studies, chloride-induced reinforcement corrosion in concrete containing metakaolin (at low replacement < 15%), calcined clay and calcined clay-limestone have been investigated for corrosion rate in chloride exposure [192, 248, 249]. Metakaolin addition at lower replacement levels of 6–20% was found to lower corrosion potential and corrosion current density in addition to delaying in the onset of corrosion initiation [248]. Similarly, corrosion rate was found to decline by nearly 50% with metakaolin addition from 0.006 mm/year to 0.004 and 0.003 mm/year with 5 and 15% metakaolin addition respectively [250]. In [192], corrosion rate of calcined clay-limestone was compared with OPC and fly ash-limestone composite blends under combined chloride-sulfate exposure conditions. The corrosion rate monitored using EIS after drying-wetting cycles by saturation at ambient temperature (25 °C) for 2 days (wetting cycles), followed by drying in a laboratory oven at 60 °C for 5 days—total cycle duration of 7 days. The results showed that calcined clay-limestone blends had lower corrosion rate initially due to higher resistivity of the cementitious matrix and the corrosion rate continued to remain lower throughout testing, as shown in Fig. 13. During the same period of testing (i.e., 16 cycles), OPC showed marked increase in corrosion rate and fly ash-limestone blends showed corrosion rate transitioning from moderate to high corrosion rate values.

For service life estimation using widespread engineering modelling tools, the significantly lower chloride transport parameter was found to ensure that concrete containing calcined clay (with > 40% kaolinite content) produced enhanced service life compared to OPC concrete [146]. Predicted service life of calcined clay-based concrete was found to be considerably longer than OPC concrete and sustainability benefit from the extended durability was evaluated using CO_2_ footprint per m^3^ normalised to the estimated service life [146]. However, when similar approach was adopted in [190] for calcined clay containing 15–20% kaolinite content the results showed only a modest reduction in the chloride transport coefficient with no significant enhancement in service life with respect to OPC with such low purity clays. This highlights that calcined clay usage in the forms of LC2 or LC3 could show variability in performance based on the kaolinite content of the clays and/or calcined clay to limestone ratio even among the clays passing reactivity threshold criteria prescribed for screening clays using rapid screening test methods [251]. Assessing suitability of SCM simply based on reactivity may not reflect the durability performance potential completely in concrete scale. The adoption of performance-based design approaches for concrete design is the best ways to enable the use of a wide range of low carbon cement formulations from the perspective of optimal utilization of all available resources.

## Case studies from field exposure studies in marine environments

Chloride ingress assessment is typically carried out in accelerated laboratory conditions as the process takes a long time in field conditions. Thus, realistic studies are quite scarce for new SCM types that are currently under development due to limited field applications. This section presents the overview of practical case studies from two marine exposure sites in Cuba [186] and India [252]. The exposure sites and the materials used based on [186, 252] are described and a practical applications of calcined clay-limestone (LC2) as mineral admixture for structures in marine exposure is highlighted from [252].

### Marine exposure sites in Cuba

**Fig. 14:**
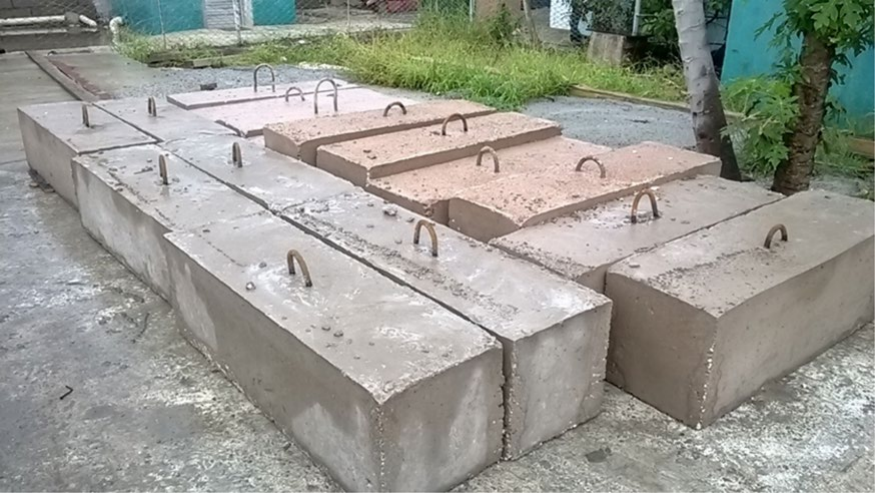
Concrete blocks produced at ECOT

To simulate the real conditions in which concrete is produced in Cuba, concrete specimens were manufactured in the premixing plant of the Empresa Constructora de Obras del Turismo, ECOT, in Cayo Santa María (CSM) (see Fig. 14). Concrete specimens were cast with a ternary blend containing 50% clinker, 30% calcined clay (original kaolinite content 48.9%), 15% limestone and 5% gypsum. A reference concrete was cast using a Type I Portland Cement, with 90% clinker, 5% limestone and 5% gypsum. Concrete blocks measuring 60 × 30 × 30 cm were prepared, which were cured in the plant for up to 28 days, and subsequently exposed at the Punta Matamoros exhibition site in July 2015. The specimens were placed and continuously monitored in natural conditions since 2015 in at least three exposure classes according to Cuban standards. Three series were cast with cement content and water-to-cement ratio following the prescriptive specifications of the Cuban standard NC 120:2014: (1) H1, less than 500 m from the seashore (high relative humidity and high chloride concentration), (2) H2, between 500 and 1500 m from the seashore (high relative humidity and mid chloride concentration), and (3) H4, more than 20 km from the seashore (mid relative humidity and low chloride concentration). The water-binder ratios of the three concretes, i.e., H1, H2 and H4 were 0.40, 0.45 and 0.55, respectively, and the detailed mix design is present in [37, 186]. Both OPC and LC3 concrete used in H1 conditions had 28 days compressive strength > 30 MPa.

**Fig. 15:**
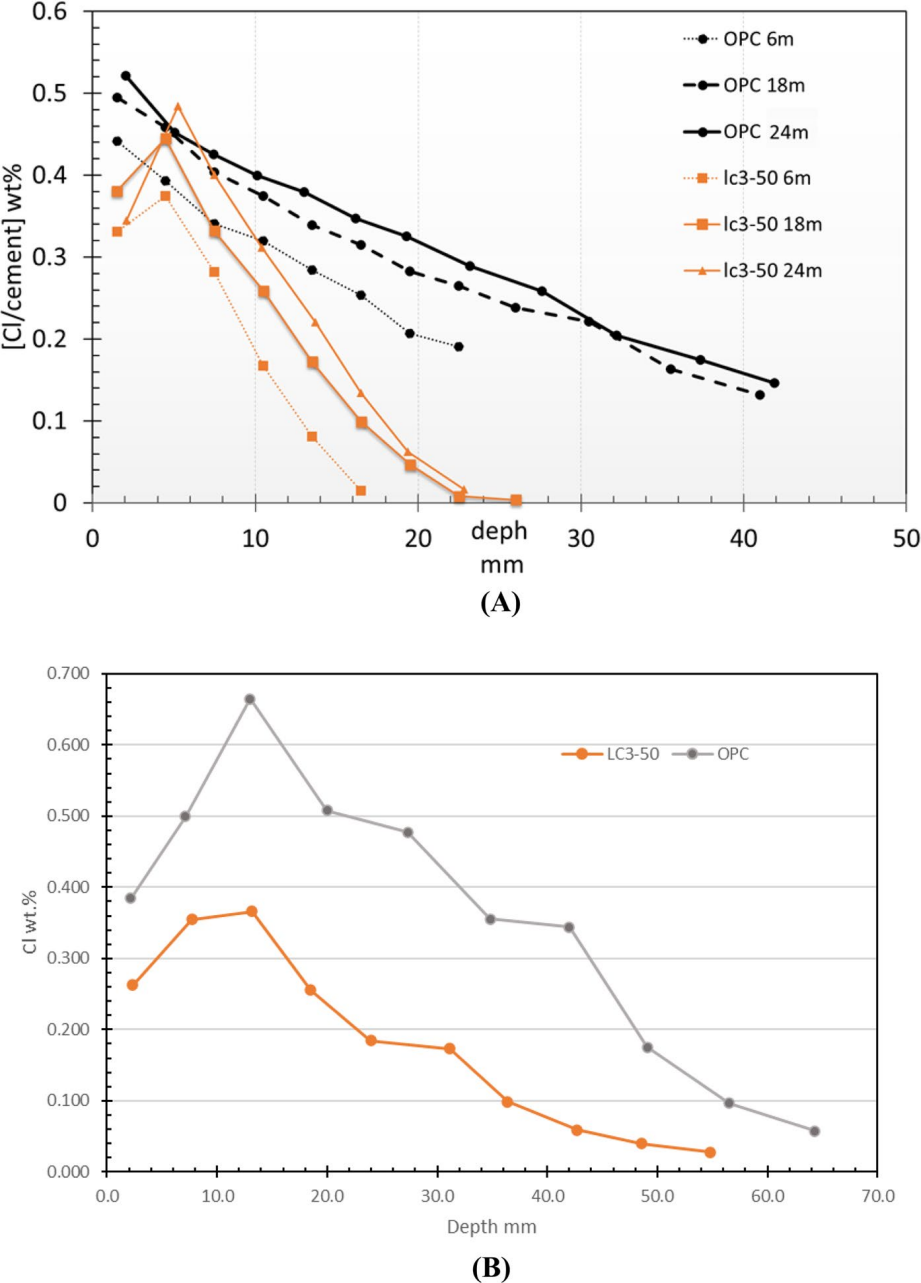
**A** Total chloride profile of mortars produced with the LC3-50 and OPC after 6, 18 and 24 months (m) of immersion in a solution with 3% NaCl. **B** Total chloride profiles of the concretes produced with LC3-50 and OPC in the ECOT CSM, after 18 months of exposure in H1 i.e., 500 m from the seashore with high relative humidity and high chloride concentration

In addition to concrete, mortars were cast with the same cement as used for concrete. The aim of the mortar study was to assess the behaviour of mortars in pure diffusion in lab conditions. The samples were immersed in a pool with a saline solution, with exposure times of 6, 12 and 23 months. The apparent diffusion coefficients were estimated, assuming that Fick's law of diffusion as per ASTM C1556 [173]. Chloride concentrations for mortars immersed in 3% NaCl solution in laboratory conditions 6, 18 and 24 months for are presented in Fig. 15A. The results are, expressed as a percentage of the total mortar weight.

Figure 15B presents the chloride profiles measured in the studied specimens. It is verified that there is a significant reduction of the total content of chlorides and the depth of penetration in the series of mortars produced with LC3-50 cement. For the same depth (for example 20 mm) the concentration of chlorides in the series produced with OPC is 7 times higher than that produced with LC3-50. This trend coincides with what is referred to in other studies carried out in which this behaviour is justified by the dense and poorly connected network of pores and increased chloride binding in the cementitious matrix of LC3-50 [104, 145], as also discussed in Sect. 2. The values of the apparent diffusion coefficients and ratio of diffusion coefficient of OPC with respect to LC3 across exposure time is shown in Table 3. At all ages, the apparent diffusion coefficient of the series produced with OPC is an order of magnitude higher than that of the series produced with LC3-50, which confirms the high capacity of the LC3 systems to inhibit the entry and migration of chlorides and other ionic species in the cementitious matrix; consistent with several laboratory observation discussion previously in Sect. 3.

**Table 3:** Values of apparent diffusivity in mortars produced with OPC and LC3-50, measured at 6, 18 and 24 months of immersion in a 3% NaCl solution

Apparent diffusion coefficient (m^2^/s)			
	6 m	18 m	24 m
LC3-50	2.46 × 10^−12^	1.39 × 10^−12^	1.19 × 10^−12^
OPC	2.62 × 10^−11^	1.25 × 10^−11^	1.34 × 10^−11^
OPC/LC3-50	10.65	8.99	11.26

Similarly, the total chloride profiles determined on the concretes exposed to field conditions (CSM series) are presented in Fig. 15B. The results show similar trend to laboratory concretes with LC3 concrete showing lower chloride content across the diffusion depth. The difference between chloride concentrations at the same depth of 20 mm is about 2.7 times compared to OPC concrete. These results confirm the beneficial use of calcined clay concrete in high chloride concentration environments.

### Marine exposure sites in India

Several demonstration structures were built with LC3 and/or calcined clay-limestone (LC2) admixture in India during the last decade [187, 252–255]. In this section, two case studies of calcined clay concrete used in marine exposure are highlighted—(1) a single storey demonstration structure built 400 m from the shore using LC2 (Calcined clay: limestone of 2:1) was used at a replacement level of 45% of the OPC and (2) Large Tetrapod concrete elements made using 30% LC2.

**Fig. 16:**
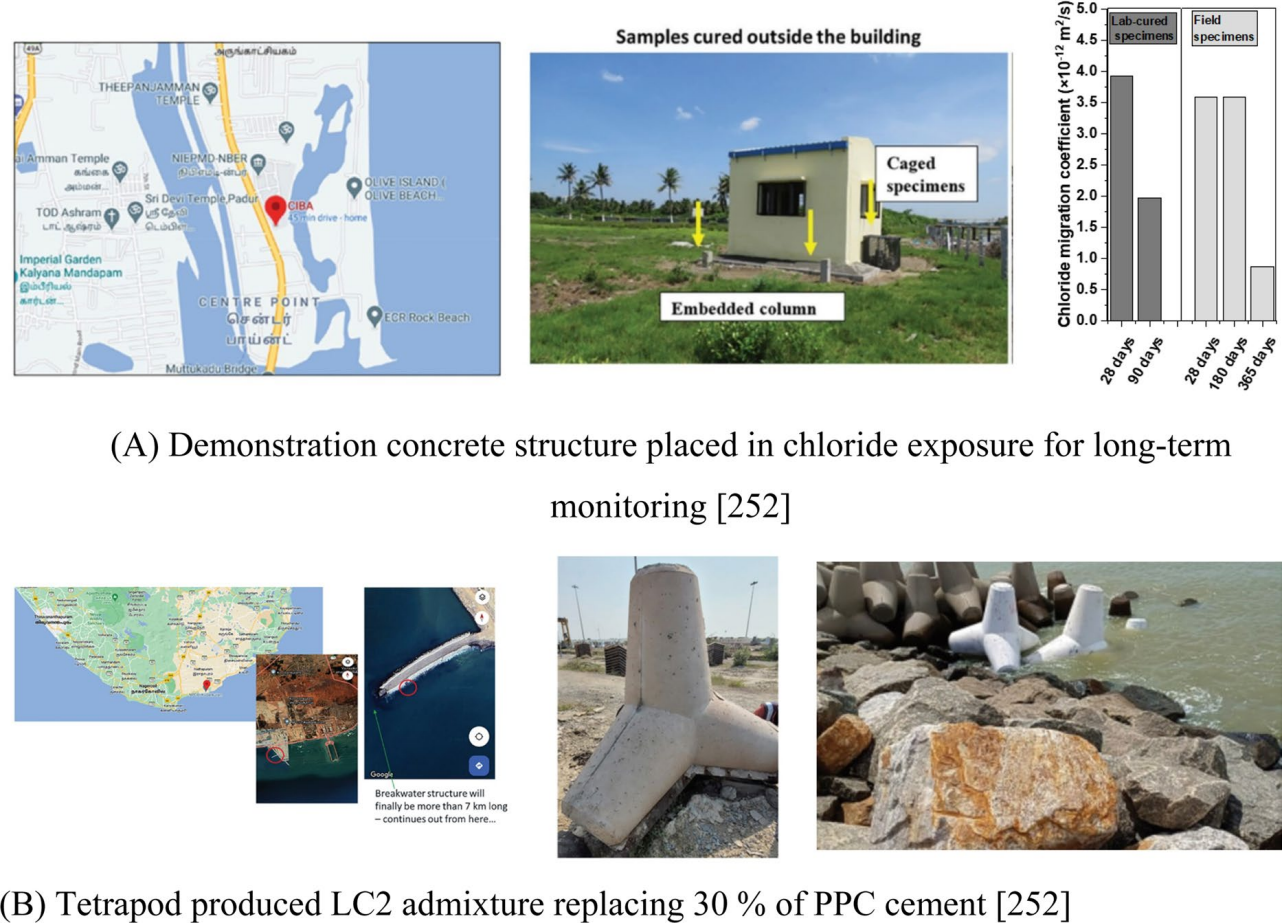
Practical use of Calcined clay-limestone (LC2) in single storey structure (**A**) and tetrapods structures (**B**) in aggressive marine environment

Figure 16A shows the locations and image of demonstration structure constructed for long-term monitoring of performance in field exposure condition. Mix design of the concrete and properties, including mechanical and durability properties, are summarised in detail [252]. Notably, the chloride resistance of the concrete used to construct the structure was characterised using non-steady state chloride migration test on concrete specimens cured in laboratory conditions and field-like conditions by placing them near the structure. The results presented in [252] are replotted and presented in Fig. 16A. Concrete had a compressive strength of 35 MPa at 28 days and the chloride migration coefficient less than 4 × 10^−12^ m^2^/s at 28 days for lab-cured concrete. The specimens placed outside in field exposure conditions showed chloride migration coefficient below 1 × 10^−12^ m^2^/s by 1 year of field exposure.

Figure 16B shows the locations and photographs of the tetrapod made with 30% LC2 admixed to PPC (Portland Pozzolana Cement, containing up to 30% fly ash). Total binder content in the concrete mixes used was 350 kg/m^3^ and water-binder ratio of 0.4 was used. The details of materials and mix design can be found in [252]. Although the LC2 was used to replace PPC which already had low clinker content (i.e., 70%), the concrete was found to attain 15 MPa after 1 day which was sufficient for the handling and shifting of the tetrapods. The concrete element placed in his natural aggressive environment will be monitored periodically for long-term performance. LC2 admixed concrete used in the structure was tested for hardened properties related to mechanical and durability performance. The charge passed in the RCPT coulombs was less than 500 and the chloride migration coefficient was about 2 × 10^−12^ m^2^/s which again confirmed the good resistance to chloride ingress in the concrete used; these values would suit most performance specifications for marine exposure concretes. Such field implementation of calcined clay showcases the promising potential in various forms i.e., LC3, LC2 or simply as calcined clay, for achieving sustainability through durability enhancement in concrete used in chloride-rich aggressive environments as shown in [146, 237, 256].

## Summary and conclusions

As calcined clay usage gains more acceptance in construction industry, the performance of calcined clay-based concrete in aggressive environments would drive the use of the materials in reinforced concrete structures that are prone to chloride-induced corrosion. Based on the detailed review of available literature dealing with chloride ingress and chemical interaction with chlorides for calcined clay based cementitious materials, the following conclusion can be drawn:

- Calcined clays can be an ideal SCM for use in chloride environments with excellent chloride resistance. This is due to a combination of factors including pore refinement, lower pore solution conductivity and changes in chloride binding characteristics. Each of these factors changes with materials characteristics such as kaolinite content of clay used, calcined clay-limestone ratio and clay type. In general, the performance of calcined kaolinite clay with about 40–50% kaolinite content is certainly improved compared to OPC, for calcined clay made with kaolinitic clay 50–70% kaolinite content, the performance is significantly improved and at lower kaolinite content below 30% availability of literature is limited to make conclusive recommendations.
- Studies on cement paste, mortar or concrete all converge to show nearly an order of magnitude lower chloride diffusion coefficient for calcined clay system, highlighting the dominant role of pore refinement in the binding matrix. Concrete containing calcined clay (with 50–70% kaolinite content in clays) is typically found to be less dependent on curing than other blended cement concrete containing fly ashes or slags. This is observed in multiple studies indicating early rise in resistivity and lower diffusion/migration coefficient at early curing age, as low as 7 days.
- Transport properties are significantly reduced compared to OPC with addition of calcined kaolinitic clays due to pore refinement while relatively fewer studies have compared lower purity kaolinite clay and other clay types. Future studies can focus on the use of lower kaolinitic content clay (< 30% kaolinite content) and alternative clay minerals to assess the potential performance obtained in comparison to calcined clays with over 40% kaolinite content. This would ensure all possible sources of clays could be utilised suitably based on their availability and for optimal utilisation of resources based on the performance requirements required in different exposure conditions.
- While some studies show synergistic development in resistivity and a marginally variation in diffusion coefficient with calcined clay-limestone ratio, there is only a modest difference in binding compared with difference in binding caused due to kaolinite content of the clay. No significant difference in performance were reported for calcined clay systems for limestone additions of 10–20%. Higher dosage may be evaluated in future to increase limestone content that could lower carbon footprint of the concrete.
- Chloride resistance in calcined clay systems is found to be similar or marginally better compared to other conventional SCMs such as fly ash and slags-based concrete. Most importantly, the performance enhancement with calcined clays occurs at early curing duration without requiring extended curing making calcined clay concretes suitable for marine concrete structures. Conventional testing methods available in concrete standards are suitable for calcined clay-based concrete.
- Calcined clay-based concretes with binary or ternary blended cementitious systems (i.e., with and without limestone) show promising results with significantly delay in the onset of corrosion, i.e., longer corrosion initiation despite reduced chloride threshold, and also lower corrosion rate which highlight the benefits of using calcined clay concrete in marine exposure conditions using existing service estimate tools utilized in concrete industry. Studies focused on chloride ingress in carbonated concrete are limited and early results show that free chlorides generated upon carbonation may influence the performance in the long term which warrants more focus in future research.
- Addition of LC2 as a performance enhancing mineral admixture with other forms of blended cements containing fly ashes/slags is also seen potential areas of accelerating uptake of calcined clay for sustainability benefits—reducing CO_2_ footprint and enhancing durability of concrete structures against chloride induced corrosion.
